# Cold Atmospheric Plasma and Plasma-Activated Water in Meat Processing: Microbial Safety, Molecular Transformations, Quality Changes, and Analytical Assessment

**DOI:** 10.3390/molecules31203697

**Published:** 2026-10-15

**Authors:** Marian Gil, Mariusz Rudy, Paulina Duma-Kocan, Renata Stanisławczyk, Dariusz Dziki

**Affiliations:** 1 Department of Agricultural Processing and Commodity Science, Institute of Food Technology and Nutrition, Faculty of Technology and Life Sciences, University of Rzeszów, Zelwerowicza 4, 35-601 Rzeszow, Poland; mgil@ur.edu.pl (M.G.); pduma@ur.edu.pl (P.D.-K.); rstanislawczyk@ur.edu.pl (R.S.); 2 Department of Thermal Technology, University of Life Sciences in Lublin, Głęboka 31, 20-612 Lublin, Poland

**Keywords:** cold plasma, plasma-activated water, advanced food analytics, meat safety, volatile organic compounds (VOCs), protein modifications, chemometrics, non-thermal technologies

## Abstract

This review describes the principles of cold plasma generation and critically evaluates the effects of cold atmospheric plasma (ACP) and plasma-activated water (PAW) on the microbial safety, physicochemical quality, and preservation of meat products, with emphasis on analytical methods used to characterize plasma-induced changes. Peer-reviewed English-language publications from 2016 to 2026 were reviewed using the Web of Science Core Collection, Scopus, PubMed, and ScienceDirect databases, with searches performed from January to June 2026. A total of 240 references supported the review. In one study conducted under comparable experimental conditions, in-package plasma treatment of diced poultry meat reduced *Listeria monocytogenes*, *Salmonella* spp., and *Escherichia coli* O157:H7 by 3.5–3.9 log CFU/cube. This study-specific range should not be generalized because antimicrobial efficacy depends on plasma-system configuration, processing conditions, target microorganism, and meat matrix. However, plasma treatment may also cause undesirable oxidative changes. In ready-to-eat ham, the TBARS value increased from 0.92 to 1.53 mg MDA/kg after treatment, indicating enhanced lipid oxidation and potential quality deterioration. ACP and PAW may improve microbial safety and extend shelf life, but treatment intensity must be carefully controlled to minimize adverse quality effects. Future research should prioritize standardized reporting, product-specific validation, toxicological assessment, industrial scale-up, regulatory compliance, and techno-economic evaluation.

## 1. Introduction

According to the Food and Agriculture Organization of the United Nations, global meat production exceeds 300 million tons annually. However, substantial losses may occur throughout the production and distribution chain because of microbial spoilage, oxidative deterioration, inadequate storage, and handling conditions. This generates drastic economic losses and poses a serious barrier to the environmental sustainability of the meat industry [1]. Meat may be exposed to microbial, chemical, and physical contamination at different stages of the production chain, including slaughter, processing, transportation, storage, and distribution. Microbial contamination is particularly relevant because it may compromise product safety and shelf life [2,3,4,5]. In addition, consumer demand for fresh and minimally processed meat products may increase the technological challenge of controlling microbial contamination while preserving sensory and nutritional quality [6]. In this review, cold plasma (CP) is used as the general term, whereas cold atmospheric plasma (CAP), also referred to as atmospheric cold plasma (ACP), denotes cold plasma generated at atmospheric pressure. High-voltage atmospheric cold plasma (HVACP or HV-CAP) refers to a high-voltage operating configuration of CAP, most commonly implemented using a dielectric barrier discharge, rather than to a separate plasma-source category.

Conventional thermal processing and chemical preservatives are effective preservation strategies; however, depending on treatment intensity, they may alter sensory attributes, nutritional quality, protein structure, and vitamin retention [7,8,9,10,11]. The situation is further complicated by the fact that some countries and regions have restricted or banned the use of certain chemicals used for food disinfection, citing the risk of residues remaining in products and possible health consequences [12]. Consequently, current research has focused on developing preservation technologies that improve microbial safety while limiting undesirable changes in the nutritional, physicochemical, and sensory properties of meat products [8,13]. Non-thermal preservation technologies include ultrasound, high hydrostatic pressure, ultraviolet radiation, pulsed electric fields, ionizing radiation, electrolyzed water, and cold plasma. These technologies may inactivate pathogenic and spoilage microorganisms while reducing heat-induced alterations in food quality [8,13,14,15,16]. Among these approaches, atmospheric cold plasma has emerged as a promising non-thermal decontamination technology for food-processing applications [6,17,18,19,20].

ACP operates at relatively low product temperatures and may reduce the need for conventional chemical sanitizers. However, its energy efficiency, economic feasibility, residue profile, and effects on sensory quality depend on the plasma configuration, treatment conditions, processing scale, and food matrix [13,21,22,23,24,25,26,27]. In meat processing, cold plasma has also been investigated for modifying surface properties, supporting brine diffusion, interacting with edible coatings, and influencing the transfer of functional compounds. However, these effects remain dependent on the plasma system and meat matrix [28,29,30,31,32,33,34].

This review aims to describe the basic principles and main systems used for cold plasma generation and to critically evaluate the effects of ACP and PAW on the microbial safety, physicochemical quality, molecular characteristics, and preservation of meat and meat products. It also examines the analytical methods used to characterize plasma-induced changes and identifies technological, toxicological, regulatory, and industrial research gaps.

Despite the increasing number of studies on ACP and PAW, comparisons among treatments remain limited by inconsistent reporting of plasma parameters, heterogeneous meat matrices, different analytical methods, and insufficient toxicological and industrial-scale validation.

## 2. Principles, Generation Systems, and Reactive Species of Cold Plasma

Plasma is commonly described as the fourth state of matter. In the cold-plasma systems considered in this review, plasma is generated directly in a gas through an electrical discharge rather than through sequential heating of a substance across the solid, liquid, and gaseous states [15,35]. The applied electric field transfers energy primarily to free electrons, which interact with neutral gas atoms and molecules. These interactions may produce excitation, ionization, dissociation, electron attachment, radicals, ions, and photons, depending on the plasma-source configuration and operating conditions [35,36,37,38,39,40].

Cold plasma is a non-equilibrium system in which electrons possess substantially more energy than the heavy particles. This energy distribution enables the generation of reactive species while limiting excessive bulk heating of the treated product under appropriately controlled conditions [36,41]. However, gas and product temperatures depend on the plasma source, applied power, working gas, treatment geometry, exposure time, and heat-transfer conditions. Therefore, the non-thermal character of the process should be experimentally verified rather than assumed [38,42,43]. Reactive species are generated mainly through electron-impact collision processes involving electrons accelerated in the applied electric field. These processes include elastic and inelastic collisions, such as excitation, dissociation, and, where relevant, ionization. In oxygen-containing plasmas, one of the key pathways for the formation of reactive oxygen species is the electron-impact dissociation of molecular oxygen, which leads to the generation of highly reactive atomic oxygen (Equation (1)) [44,45]:

e^−^ + O_2_ → 2O + e^−^ (1)

In oxygen-containing plasma systems, electron-impact dissociation of molecular oxygen may generate atomic oxygen, which can subsequently participate in ozone formation through three-body collisions. The relevance of these reactions depends on pressure, humidity, gas composition, discharge configuration, and operating conditions [44,45,46]. The profile of plasma-generated reactive species depends on the working gas, humidity, electrical conditions, discharge geometry, and interaction with the treated matrix. In plasma-treated water, these factors influence pH, oxidation-reduction potential, and the concentrations of H_2_O_2_, NO_2_^−^, and NO_3_^−^ [46,47,48,49,50]. These nitrogen-reaction pathways provide a mechanistic basis for the in situ generation of nitrite and nitrate during plasma-assisted curing. However, the concentrations and balance of these species also depend on the plasma-source configuration, humidity, treatment time, temperature, gas–liquid or gas–meat interfacial transfer, and matrix composition [47,48,50].

Cold plasma may be generated using ambient air, oxygen, nitrogen, carbon dioxide, hydrogen, noble gases, or controlled gas mixtures. Noble gases can facilitate discharge ignition and provide a controlled background for the addition of reactive gases, whereas air-based systems may reduce gas-related operating costs [51,52]. Appropriately designed air-based systems can also provide stable and reproducible operation, while avoiding the need for a continuous supply of noble gas.

Feed-gas selection should be regarded as an application-specific variable rather than a universal determinant of plasma stability or treatment effectiveness. Selection should consider microbial efficacy, reactive-species generation, oxidation risk, product characteristics, gas consumption, process complexity, and operating cost [53,54].

Plasma sources used in food processing include dielectric barrier discharges [15,55,56,57,58,59,60], corona and pulsed-corona systems [15,43,57,58,61], atmospheric-pressure plasma jets [39,40,43,55,56,58,59,61,62,63,64,65], radio-frequency systems, and microwave-driven sources. Gliding-arc systems should be classified as cold plasma only when operation under non-thermal conditions has been experimentally demonstrated [37,57].

In the food industry, cold plasma is primarily generated using dielectric barrier discharges, corona discharges, atmospheric plasma jets, gliding discharges, and glow discharges. Systems operating at atmospheric pressure are the most important in terms of applications, as they do not require the use of vacuum chambers and can be relatively easily integrated into production lines. DBD sources, enabling the treatment of large product surfaces, and plasma jets, ensuring precise local interaction, are particularly common. The source selection depends on the product type, its geometry and composition, the required process efficiency, the type of working gas, and the allowable changes in the sensory and chemical properties of the food [66]. A summary of plasma sources, typical electrical excitation, and application modes is provided in Table 1.

Dielectric barrier discharge (DBD) is one of the most commonly used cold plasma sources in food technology. The system typically consists of two electrodes, at least one of which is coated with a dielectric that limits the current flow, prevents the formation of an electric arc, and enables the generation of short-duration microdischarges [67]. DBD can operate at atmospheric pressure, also using air as the working gas, thus eliminating the need for a vacuum chamber. It enables the treatment of large surfaces and the decontamination of food and packaging materials, even directly within a closed package [66,67,68]. The main advantages of this technology include its simple design and the possibility of integration into production lines. Its limitations include the uneven processing of products with complex shapes and the risk of local heating and surface oxidation.

A corona discharge is generated in a non-uniform electric field, typically between a pointed and a flat electrode, by ionizing a gas without creating an electric arc. It can be generated in air at atmospheric pressure and used for food decontamination, air treatment, and packaging modification [68]. The advantages of this source include its simple design and low energy consumption, while its limitations include a small operating area, uneven processing of complex surfaces, and the formation of ozone and nitrogen oxides.

Atmospheric-Pressure plasma jet (APPJ) transports reactive components from the nozzle to the product surface using a gas such as helium, argon, nitrogen, or air. It enables precise decontamination of small and irregular surfaces, even in indirect mode. Limitations include the small operating area and the cost of noble gases, which make the technology difficult to scale.

Gliding arc discharge moves between divergent electrodes under the influence of a gas stream. It produces reactive oxygen and nitrogen species, which is why it is used for surface decontamination and water activation. A limitation is the increase in gas temperature, which requires control when processing heat-sensitive foods.

Glow discharge produces a homogeneous plasma at reduced pressure, induced by a direct current, alternating current, or radio frequency field. It is used to decontaminate dry products, seeds, spices, and packaging. However, the need for a vacuum chamber increases costs and complicates integration into production lines [67].

Radio and microwave sources generate cold plasma by contactlessly transferring energy to electrons, ensuring a stable discharge and reducing the risk of electrode contamination. They are used for decontaminating packaging and equipment, processing bulk products, and activating gases and liquids. Their use is limited by the complexity and cost of the equipment and the need for electromagnetic radiation shielding.

Regardless of the cold source design, plasma can be used in several modes. In direct treatment, the product is placed in the discharge area, where it is exposed to reactive components, charged particles, an electric field, and radiation. An alternative is indirect treatment, in which the product remains outside the discharge area and is primarily exposed to reactive components transported by gas. An extension of this approach is in-pack treatment, which involves generating plasma in the gas space of a closed package, reducing the risk of product recontamination and potentially extending its shelf life [66]. Yet another variant of indirect treatment is the use of a plasma-activated liquid containing reactive oxygen and nitrogen species, which can be used for cleaning, disinfection, or treatment of the product.

Depending on electrode geometry, dielectric properties, and operating conditions, DBD systems may operate in diffuse, filamentary, or mixed discharge modes. Gas flow may affect filament distribution in flow-assisted systems; however, discharge behaviour and stabilization result from the combined effects of the electrical parameters, reactor configuration, working-gas properties, and surrounding conditions. Therefore, plasma-treatment intensity should not be characterized by voltage alone. Studies should report the voltage waveform, current, frequency, pulse duration and repetition frequency, absorbed power, electrode geometry, discharge gap, dielectric material and thickness, working-gas composition, humidity, gas flow rate, and treatment time. Because the power delivered to the plasma depends on the time-dependent relationship between voltage and current, it should be determined from appropriate electrical measurements rather than inferred solely from the applied voltage [69]. These parameters collectively determine the discharge mode, power deposition, reactive-species profile, and intensity of plasma treatment.

CP treatment may be applied using direct, remote, non-contact, indirect, or in-package configurations. These terms describe the relationship between the plasma source and the treated product rather than the physical mechanism used to generate the discharge. In direct treatment, the product is exposed to the active discharge region or its immediate vicinity. In remote treatment, the discharge is generated away from the product, and the plasma effluent and transported reactive species reach the treated surface. In-package treatment involves plasma generation within the package headspace, whereas indirect treatment may involve a plasma-activated carrier, such as water, brine, or another solution, that is subsequently applied to the product. Plasma-activated water, plasma-treated brines, and plasma-activated powders should therefore be considered indirect application routes or activated carriers rather than separate plasma-source types. Similarly, plasma-assisted curing and combinations of CP with modified-atmosphere packaging, edible coatings, essential oils, UV treatment, or other preservation processes represent technological applications or hybrid processing strategies rather than independent categories of plasma generation.

The source-to-sample distance, working-gas composition, surrounding atmosphere, and treatment configuration influence the identity, concentration, lifetime, and transport of reactive species reaching the treated material [66,70]. These factors are particularly important in remote and in-package systems because reactive species may undergo recombination, transformation, interaction with packaging materials, or secondary reactions with water vapour and other headspace constituents before reaching the product.

In cold plasma technology, “dose” cannot be adequately defined by a single parameter. The effective treatment intensity results from the combined effects of the power delivered to the discharge, exposure time, source-to-sample distance, working-gas composition and flow, humidity, treatment area, discharge mode, surrounding atmosphere, and product geometry [71]. For in-package applications, package volume, headspace composition, packaging-material properties, and the delay between plasma generation and microbiological or physicochemical assessment should also be reported. These interacting parameters determine the fluxes of reactive oxygen and nitrogen species, charged particles, photons, and electric fields reaching the treated surface and consequently govern the biological and physicochemical response [72,73]. Changes in source-to-sample distance may substantially alter both the concentrations and relative proportions of the delivered reactive species because short-lived species may decay or react during transport, whereas longer-lived species may persist over greater distances [74]. Therefore, comparisons between CP studies should not be based solely on the plasma-source name, applied voltage, or exposure time. For reproducibility and meaningful cross-study comparison, future studies should report voltage, current, frequency, waveform, absorbed power, exposure time, source-to-sample distance, treatment area, product dimensions and matrix geometry, working-gas composition and flow rate, humidity, and sample temperature. For in-package applications, package and headspace volume, the product-to-headspace volume ratio, packaging-material properties, headspace composition, and the interval between plasma generation and microbiological or physicochemical assessment should also be specified.

To avoid combining different classification levels, the CP systems discussed in this review are presented in two complementary tables. The first (Table 2A) classifies the physical plasma-source types, whereas the second summarizes the treatment layouts, plasma-activated carriers, and technological application modes used in meat processing (Table 2B). HVACP/HV-CAP is not presented as a separate plasma-source category because it describes a high-voltage atmospheric operating configuration that may be implemented using different source and application designs.

Available evidence supports the technical feasibility of cold plasma at the laboratory scale [108]; however, industrial performance, process reproducibility, and economic feasibility remain insufficiently established.

## 3. Mechanisms of Microbial Inactivation

Cold-plasma-mediated microbial inactivation results from the combined action of reactive oxygen and nitrogen species, charged particles, electric fields, and, in some systems, ultraviolet radiation [37,43,51,109,110].

The first mechanism involves the interaction of reactive oxygen species (ROS), such as ^1^O_2_, O_3_, O_2_•^−^, •OH, and H_2_O_2_, and reactive nitrogen species (RNS), such as NO, NO_2_, ONOO^−^, NO_3_^−^, and NO_2_^−^, with microbial cells [59,66,110,111]. Plasma-generated ROS and RNS may disrupt microbial redox homeostasis and induce oxidative stress, damaging cell walls, cytoplasmic membranes, membrane lipids and proteins, enzymes, and intracellular components. Oxidation of membrane lipids and proteins may increase membrane permeability and promote the leakage of intracellular constituents, including proteins and genetic material, ultimately contributing to microbial inactivation [22,66,111,112]. Although ACP can inactivate both Gram-negative and Gram-positive bacteria, their susceptibility and dominant inactivation pathways may differ because of variations in cell-envelope structure [22,112]. However, susceptibility cannot be attributed to Gram classification alone, as it also depends on the physiological state of the cells, plasma-source configuration and operating conditions, treatment time, and the composition and structure of the surrounding food matrix. In bacterial spores, ROS and RNS may oxidatively damage the spore coat and other protective structures, whereas plasma-generated electric fields may independently induce membrane permeabilization or electroporation. Oxidative damage and electroporation should therefore be regarded as distinct but potentially complementary mechanisms of spore inactivation [22,38].

The second mechanism involves plasma-generated photons, including UV radiation. However, the contribution of UV to microbial inactivation is source- and configuration-dependent and should not be generalized across cold-plasma systems. Accordingly, UV radiation cannot be regarded as a universal major inactivation factor in cold-plasma meat-treatment applications. Its relative contribution depends on the plasma-source type, emission spectrum and intensity, treatment distance, exposure geometry, treatment time, direct or remote configuration, and the optical and structural properties of the meat matrix. In the near-field plasma configuration investigated by Gierke et al. [113], UVA and UVB had negligible antimicrobial effects, whereas UVC was estimated to contribute under the specific experimental conditions. Similarly, Salgado et al. [114] found that UV enhanced the inactivation of *Escherichia coli* biofilms exposed to a surface barrier discharge through synergistic interactions with plasma-generated long-lived reactive species rather than through direct UV-induced DNA damage alone. In many remote or indirect plasma systems, long-lived reactive species, including O_3_ and other reactive oxygen and nitrogen species, may therefore contribute more substantially to microbial inactivation than direct UV exposure [36,113,114]. Their diffusion may also reduce shadowing effects on complex three-dimensional food surfaces compared with conventional UV decontamination [38]. However, treatment with direct and spatially localized plasma sources, including plasma jets, may remain susceptible to shadowing. Consequently, the contribution of UV radiation relative to reactive species should be experimentally verified for each plasma source, configuration, treatment distance, and food matrix [113,114]. The third mechanism involves oxidative damage to intracellular macromolecules after reactive species or secondary oxidative processes reach intracellular targets. Plasma exposure may damage DNA, proteins, and polysaccharides, thereby impairing essential metabolic functions and replication processes and ultimately contributing to microbial inactivation. This pathway is distinct from the first mechanism, which primarily concerns oxidative damage to the cell envelope and membrane-associated components [66,110,111,115]. Owing to its non-equilibrium nature, cold plasma may operate with a relatively low bulk-gas temperature and thus limit heat-related changes in food. However, preservation of nutritional and sensory quality requires adequate monitoring and control of both gas and product temperatures throughout treatment [13,116]. Process effectiveness depends on the microbial species and contamination level, plasma-system configuration and operating parameters, and food characteristics, including water activity, protein and lipid content, and the morphological and structural properties of the food matrix [22,37,38]. Most food-related ACP studies have focused on bacterial pathogens, including *Escherichia coli*, *Salmonella* spp., and *Listeria monocytogenes*, associated with meat, dairy products, and vegetables [117,118]. Although ACP has also been investigated against viruses, yeasts, fungi, and bacterial spores, the available evidence is more strongly dependent on the plasma configuration, treatment medium, microbial characteristics, and experimental conditions [22,118]. In the case of *Saccharomyces cerevisiae*, treatment effectiveness varied among media, with greater inactivation observed in aqueous and saline environments, which was attributed to increased •OH-radical generation under the investigated conditions [118].

The effects of cold-plasma process parameters on microbial inactivation and meat quality are summarized in Table 3.

## 4. Application of Cold Plasma in Meat and Meat Products

ACP and PAW have been investigated as non-thermal strategies for microbial control and shelf-life extension in meat products [60,105,124,125,126]. However, treatment uniformity and effectiveness depend on product geometry, surface irregularities, plasma-source configuration, and exposure conditions. The practical applications of ACP and PAW in different categories of meat products, together with the reported effects and the most common quality risks, are summarized in Table 4.

### 4.1. Applications in Poultry Meat

Cold plasma (CP) treatment has been extensively studied for poultry preservation due to its potential to reduce microbial contamination and extend shelf life under refrigerated conditions [85,130]. However, the process’s efficacy depends on the plasma source, operating parameters, process gas, sample geometry, target microorganism, initial contamination level, and storage conditions. Moutiq et al. [83] subjected cylindrical chicken breast samples (23 mm diameter, 10 g mass)—placed in 168 mm × 120 mm × 28 mm polypropylene containers and sealed with high-barrier polypropylene film—to a dielectric barrier discharge (DBD) cold atmospheric plasma system in dry air (<5% relative humidity). Plasma was generated at 100 kV and 60 Hz for 1, 3, and 5 min. Subsequently, the samples were stored under aerobic conditions at 4 °C for 24 days. Initial counts for mesophilic bacteria, psychrotrophic bacteria, and Enterobacteriaceae in untreated samples were 4.5 log CFU/g, 4.6 log CFU/g, and 3 log CFU/g, respectively; following a 5-min treatment at 100 kV and 24 h of storage, these counts decreased to 2.6 log CFU/g, 3 log CFU/g, and 1.3 log CFU/g. After 24 days, the counts of these microbial groups in the plasma-treated samples were approximately 1.5, 1.4, and 0.5 log CFU/g, respectively [83]. Thus, at the end of the storage period, differences of approximately 3.0, 3.2, and 2.5 log CFU/g, respectively, were observed in the treated samples compared to the control samples.

In a study by Zhuang et al. [84] on chicken breast processing [85], two chicken tenderloin fillets (each weighing approximately 90.6 ± 6.5 g) and two cylindrical muscle samples (2.5 cm in diameter, 2.5–3.0 cm thick, and weighing 21 ± 1.5 g) were placed on polymer trays (19.5 cm × 14.5 cm × 4.0 cm) and sealed with polypropylene film. The packaged samples were subjected to dielectric barrier discharge (DBD) treatment at 70 kV for 60, 180, or 300 s. Control samples were packaged identically but not exposed to plasma and were stored at 4 °C for 5 days. A 60-s CP treatment reduced final populations of psychrophiles by 0.52 log CFU/ml, *C. jejuni* by 0.79 log CFU/ml, and *Salmonella* Typhimurium by 0.41 log CFU/ml. Extending the treatment time from 60 to 180 s increased the reduction in the psychrophile population by an additional 0.63 log CFU/ml (from 7.81 to 7.18 log CFU/ml). Further extending the exposure to 300 s did not improve the effect, as the psychrophile count was 7.33 log CFU/ml. For packaged, diced cooked chicken breast (1.5 × 1.5 × 1.5 cm, approx. 3.8 g) coated with whey protein, an in-package cold plasma system utilizing dielectric barrier discharge (DBD) at atmospheric pressure was employed, operating at 39 kV for 3.5 min [131]. The plasma was generated in ambient air. The effect on *Salmonella*, *Escherichia coli* O157:H7, and *Listeria monocytogenes* was investigated. Following the process, microbial counts decreased by 3.7 log CFU/cube for *Salmonella*, 3.9 log CFU/cube for *E. coli* O157:H7, and 3.5 log CFU/cube for *L. monocytogenes* [131]. Rossow et al. [62] treated chicken breast skin and meat with cold plasma using various feed gases (argon, air), exposure times (30–180 s), and distances between the plasma source and the sample (5, 8, or 12 mm) at a voltage of 2–3 kV and a frequency of 1 MHz. The effect on *Campylobacter jejuni* was investigated, with an initial contamination level of 10^6^–10^7^ CFU/cm^2^. The maximum mean reduction in *C. jejuni* using argon or air as feed gases ranged from 0.78 to 2.55 and from 0.65 to 1.42 log CFU/cm^2^, respectively. Application of argon plasma for 120 s at a distance of 8 mm resulted in a significant increase in the lightness of chicken breast fillets. Since the surface temperature reached a maximum of 61 °C, the contribution of thermal denaturation to the process of microbial inactivation and color changes cannot be ruled out.

Zhuang et al. [82] optimized in-package dielectric barrier discharge cold plasma treatment of raw chicken breast using response surface methodology. The effects of voltage (60–80 kV), exposure time (60–300 s), and modified-atmosphere composition (35–95% O_2_, 0–60% CO_2_, and approximately 5% N_2_) were evaluated after 5 days of storage at 4 °C. The initial populations of psychrotrophs, *Campylobacter jejuni*, and *Salmonella enterica* ser. Typhimurium were 5.01, 5.06, and 5.40 log_10_ CFU/ml, respectively. The lowest psychrotrophic count, 5.91 log_10_ CFU/ml, was obtained after treatment at 60 kV for 180 s in 35% O_2_/60% CO_2_/5% N_2_, compared with 7.70 log_10_ CFU/ml in the air-packaged treatment. The lowest counts of *C. jejuni* and *Salmonella* Typhimurium were 3.36 and 4.35 log_10_ CFU/ml, respectively. However, voltage, treatment time, and modified-atmosphere composition did not significantly affect the two inoculated pathogens. IPCP had no significant effect on pH or drip loss, but altered meat color depending on interactions among treatment time, voltage, and oxygen content. A modified atmosphere containing 35% O_2_, 60% CO_2_, and 5% N_2_ combined with treatment at 60 kV for 60 s was recommended as the most favorable compromise for limiting psychrotrophic growth and minimizing changes in meat appearance.

Gao et al. [105] investigated the combined effect of cold plasma and rosemary. Ground chicken breast patties weighing 15 g, measuring 50 mm in diameter and 5 mm in thickness, were prepared with or without 1% rosemary extract and packaged under a modified atmosphere containing approximately 63% O_2_ and 31% CO_2_. The packaged samples were directly treated using in-package dielectric barrier discharge cold plasma at 70 kV for 180 s and stored at 4 °C for 5 days. Untreated samples packaged under the same atmosphere served as controls. DBD-CP did not significantly reduce the total aerobic count immediately after treatment but inhibited microbial growth during refrigerated storage. After 5 days, the total aerobic count was 3.75 log CFU/g in plasma-treated samples compared with 4.53 log CFU/g in untreated controls, representing a difference of 0.78 log CFU/g. However, plasma treatment accelerated lipid oxidation, increasing TBARS to 2.48 mg MDA/kg after storage, compared with 0.93 mg MDA/kg in untreated samples. Rosemary extract reduced microbial counts and limited lipid oxidation. After 5 days, rosemary decreased TBARS by approximately 0.84 mg MDA/kg in plasma-treated patties. DBD-CP also decreased pH and the a* and b* color coordinates, whereas rosemary darkened the product and increased redness and yellowness. The results indicate that rosemary extract can partially counteract plasma-induced lipid oxidation while contributing to the microbiological stability of ground chicken meat.

### 4.2. Applications in Beef and Pork

Beef, classified as red meat, is characterized by a high lipid content, which, combined with microbial activity, increases its susceptibility to spoilage. However, the possibility of effective microbial reduction in packaged beef without compromising its quality properties has been reported under the evaluated experimental conditions [44]. The use of ACP in a modified atmosphere (30% O_2_ + 70% Ar) enabled a 1.6-log reduction in *Listeria innocua* in RTE beef [132], and effectiveness against *Listeria monocytogenes* has also been demonstrated for ham [128], dried beef [126], fresh pork, and bresaola [132]. Studies on beef bastirma have shown that the use of ACP at different exposure times and gas compositions led to a significant reduction in the number of *Staphylococcus aureus* and *Listeria monocytogenes* from 5.78 and 5.71 log CFU/cm^2^ to 0.85 and 0.83 log CFU/cm^2^, respectively [133]. Additionally, the combination of atmospheric plasma with clove oil showed a synergistic effect against *E. coli* and *Staphylococcus aureus* in beef jerky, achieving a 7.5 log reduction [106].

### 4.3. Plasma-Assisted Curing and Modification of Meat Products

Plasma–liquid interaction can generate reactive oxygen and nitrogen species and decrease the pH of the liquid [134,135,136]. In air or other nitrogen- and oxygen-containing plasma systems, gas–liquid transfer may involve NO_2_, its dimer N_2_O_4_, N_2_O_5_, HONO, and HNO_3_, depending on their gas-phase abundance, Henry’s law partitioning or effective solubility, interfacial uptake, and chemical reactivity [134,137,138]. Direct dissolution of NO should not be regarded as the principal universal pathway of nitrite and nitrate formation because its physical solubility in water is limited and its transfer competes with gas-phase oxidation and interfacial reactions [137,138]. Moreover, the uptake of reactive nitrogen oxides may deviate from non-reactive Henry’s law equilibrium because dissolution is coupled with hydrolysis, acid dissociation, and secondary aqueous reactions [134,137,138]. Interfacial uptake and hydrolysis of higher nitrogen oxides can contribute to the formation of nitrous acid, HNO_2_/HONO, and nitric acid, HNO_3_. These acids establish aqueous acid–base equilibria with their conjugate bases, nitrite, NO_2_^−^, and nitrate, NO_3_^−^, respectively. Therefore, liquid acidification results from the formation and dissociation of nitrous and nitric acids and from other proton-generating reactions, whereas NO_2_^−^ and NO_3_^−^ are conjugate bases and should not be described as acidic species. The dominant pathways depend on the plasma source, gas composition, temperature, liquid pH and composition, gas–liquid interfacial area, and mass-transfer conditions [134,135,136,137,138]. During plasma-assisted curing, the reactive nitrogen species generated in the gas and liquid phases can lead to the in situ formation of nitrite and nitrate. These species may participate in cured-color development and contribute to microbial control, including the inhibition of *Clostridium botulinum*. However, the concentrations of nitrite and nitrate generated by direct plasma treatment or delivered through PAW are strongly dependent on the meat matrix and processing conditions and should not be generalized across different meat products. Their formation, transfer, retention, and depletion may be influenced by the plasma-source configuration, working-gas composition, treatment intensity, PAW properties, product-to-liquid ratio, meat pH and water activity, fat and protein content, curing ingredients, storage conditions, and subsequent thermal processing. Initial and residual nitrite and nitrate concentrations should therefore be quantified separately for each meat product and processing system, together with a product-specific assessment of N-nitrosamine formation during storage and after cooking. Accordingly, although DBD treatment may eliminate the need for the direct addition of conventional curing additives such as sodium nitrite (E250) or potassium nitrite (E249), the resulting process should not be described as “nitrite-free”. More accurately, it represents curing without directly added nitrite or nitrate, in which curing-active nitrogen species are generated in situ during plasma treatment. This approach may support the development of clean-label meat products, although the use of this claim depends on the applicable regulatory requirements and on the residual concentrations of nitrite and nitrate in the final product. However, its use is still largely limited to research and pilot applications, and further work is necessary to optimize process parameters, quantify the formation and depletion of nitrite and nitrate, ensure product safety and quality, and support potential industrial-scale implementation [99]. The potential of plasma-assisted curing without directly added nitrite has been demonstrated in dried pork [129] and canned minced ham [63,139]. Nevertheless, these products should not be regarded as chemically nitrite-free if measurable amounts of nitrite or nitrate are generated during treatment. In the case of canned minced ham cured with DBD ACP, no deterioration in sensory characteristics in terms of taste and aroma was observed compared to conventionally cured products, and in some samples their improvement was noted [63]. Nevertheless, sensory acceptance alone is insufficient to establish the overall suitability and safety of plasma-assisted curing. A comprehensive assessment should also include residual nitrite and nitrate concentrations, the formation and stability of cured-meat pigments, microbial safety, including the control of *Clostridium botulinum*, and the potential formation of N-nitrosamines. These parameters should be monitored not only immediately after treatment but also during storage and after cooking, because reactive nitrogen species, curing pigments, microbial populations, and potentially hazardous reaction products may change over time or during thermal processing. Although plasma curing may contribute to desirable sensory attributes and microbial control under optimized conditions, these effects are highly dependent on treatment parameters and food matrix composition. Therefore, conclusions regarding sensory quality and the inhibition of *Clostridium botulinum* should be interpreted as product- and process-specific rather than universally applicable.

Alternatively, the use of PTW/PAW as a curing medium led to comparable or slightly better color-forming effects, provided that the nitrite concentration was balanced [89,120]. However, color development should be distinguished from pigment stability during storage and cooking. Consequently, measurements of instrumental color and curing pigments should be supplemented with an assessment of residual nitrite and nitrate and their changes throughout the product shelf life. A study on smoked pork sausage compared products made without sodium nitrite (negative control), products cured with sodium nitrite (positive control), and products made using plasma-activated solutions of soy, pea, and lentil proteins, employing “electronic nose” analysis and GC-IMS techniques [96]. Sausages cured with ACP-treated brine showed an increased concentration of 2-methylbutanal, formed as a result of isoleucine degradation in the Strecker reaction, characteristic of high-quality cured Jinhua hams or Iberian tenderloin [96,140]. Sensory evaluation of smoked pork sausage did not reveal any significant differences between the products in terms of smoked taste and overall acceptability [96]. These findings indicate that ACP-treated brine did not adversely affect the sensory acceptance of the tested sausage formulation. However, they do not, by themselves, demonstrate equivalent chemical or microbial safety. Such a conclusion would require parallel determination of residual nitrite and nitrate, relevant vegetative microorganisms and bacterial spores, and volatile and non-volatile N-nitrosamines during storage and after cooking.

PAW and electrolyzed water were also reported to limit the growth of bacteria, fungi, and yeasts in beef while increasing surface lightness and altering other color parameters [91]. Furthermore, the use of plasma-activated brine increased the a* value of dried beef without significantly affecting texture and lipid oxidation, which was explained by the formation of nitrosylmyoglobin and nitrosylmyochromogen [92]. Nevertheless, an increase in redness at a single analytical time point should not be interpreted as evidence of long-term pigment stability. Evaluation of plasma-assisted curing should therefore include changes in instrumental color, nitrosyl pigments, metmyoglobin formation, lipid oxidation, residual nitrite and nitrate, microbial counts, sensory acceptance, and N-nitrosamine concentrations throughout storage and following cooking. Overall, the available results indicate the potential of plasma-assisted curing, but direct comparisons with conventional curing require harmonized and product-specific evaluation of technological performance, chemical safety, microbial safety, and sensory quality.

## 5. Effects of Cold Plasma on the Physicochemical, Functional, and Sensory Properties of Meat

### 5.1. Molecular Modifications of Meat Biopolymers and Components

From the perspective of the chemical composition of muscle products, proteins—next to water—constitute the dominant structural and functional fraction. Their molecular environment is characterized by the simultaneous presence of unsaturated lipids, reactive carbohydrates, and metal ions with catalytic potential, which makes muscle proteins particularly susceptible to chemical and conformational modifications under the influence of CP [141]. Plasma-generated ROS and RNS may induce protein oxidation through several interconnected pathways, including carbonyl formation, oxidation of sulfhydryl groups, aggregation, fragmentation, and alterations in protein secondary and tertiary structures. However, the direction and magnitude of these changes depend on the plasma dose, the amino acid and protein composition, and the physicochemical characteristics of the food matrix. The high reactivity of ROS/RNS towards susceptible amino acid residues, especially cysteine, promotes the oxidation of sulfhydryl groups and the formation of disulfide bonds, leading to protein cross-linking and aggregation [142]. Controlled CP-induced oxidation may promote limited cross-linking of myofibrillar proteins, thereby modifying gel-network formation and the rheological and techno-functional properties of meat products [143,144,145]. Conversely, more extensive oxidation may result in excessive aggregation or protein-chain fragmentation, with consequent deterioration of protein solubility and functionality [146,147].

Under more intense CP treatment conditions, increased muscle protein oxidation is observed, manifested by a decrease in the thiol group content and an increase in the carbonylation level [143,148,149], as well as the formation of dityrosine in myofibrillar proteins [144]. These changes are directly related to a decrease in the solubility and an increase in the size of protein aggregates [146]. Depending on the treatment intensity and the susceptibility of individual protein fractions, oxidative modifications may promote either cross-linking and aggregation or peptide-bond cleavage and fragmentation. Therefore, aggregation should not be regarded as the only possible structural outcome of CP treatment. At the structural level, CP induces a reorganization of secondary and tertiary structures, typically characterized by a reduction in the alpha-helix content with a simultaneous increase in beta-sheets and disordered structures, indicating partial unfolding of the protein conformation [150]. Nevertheless, the extent and direction of these structural transitions are not universal and may vary according to plasma dose, protein type and composition, and matrix characteristics, including pH, water activity, lipid content, ionic strength, and the presence of endogenous antioxidants or pro-oxidant metal ions. The accompanying changes in the surface hydrophobicity of myofibrillar proteins should be interpreted in relation to the plasma dose and the characteristics of the treated matrix. At low to moderate plasma doses, reactive oxygen and nitrogen species may induce partial unfolding of myofibrillar proteins, exposing previously buried hydrophobic residues and thereby increasing surface hydrophobicity [151,152,153]. At higher treatment intensities, however, extensive oxidation, aggregation, fragmentation, or rearrangement of hydrophobic regions may produce a different or even opposite response. This effect may contribute to improved protein functionality, including enhanced protein–protein interactions and gel network formation. Mild plasma-induced structural modifications may also improve selected techno-functional properties, including emulsification and water retention, by exposing reactive and hydrophobic residues and facilitating interactions between protein molecules and between proteins, water, and lipids. However, once the optimal dose range is exceeded, further oxidation and denaturation may promote intermolecular cross-linking and aggregation of unfolded protein molecules. Excessive treatment may consequently reduce protein solubility, impair protein hydration and interfacial activity, and ultimately decrease gel-forming, emulsifying, and water-holding performance. Under these conditions, hydrophobic groups may become buried within aggregates or become less accessible to hydrophobicity probes, resulting in a decrease in measured surface hydrophobicity [147]. Therefore, the reported increases and decreases in surface hydrophobicity should not be regarded as contradictory, but rather as different stages of dose-dependent plasma-induced protein modification. The transition from desirable protein unfolding to deleterious aggregation may be considered a dose-dependent “tipping point”, whose position depends on voltage, exposure time, gas composition, plasma configuration, protein composition, and the physicochemical properties of the food matrix. DBD ACP treatment also affects the volatile aldehyde profile by modifying the structure of myofibrillar proteins [154]. In myofibrillar proteins extracted from dry-cured bacon, a decrease in the concentration of five aldehydes in the headspace has been observed, especially at higher voltages (60 and 70 kV) [154]. However, changes in headspace aldehyde concentrations may reflect not only structural modifications of proteins but also altered protein–volatile interactions and changes in aldehyde formation, retention, or release. Therefore, such changes should not be interpreted solely as direct evidence of improved protein functionality or sensory quality.

These modifications are crucial for the techno-functionality of muscle proteins, but their effects should be considered in the context of a clear compromise between functionality and the sensory quality of the final product. At relatively mild treatment intensities, partial protein unfolding and limited oxidation may improve gel formation, emulsifying properties, and water retention. In contrast, excessive oxidation may cause extensive denaturation, intermolecular cross-linking, aggregation, fragmentation, and loss of solubility, resulting in reduced functional performance. Thus, the effects of CP cannot be classified as beneficial or detrimental solely on the basis of treatment time or applied voltage, but should be evaluated using protein-specific structural and functional indicators.

In this review, “moderate plasma exposure” is used to describe system-specific experimental conditions associated with increased surface hydrophobicity and limited structural modification, such as 5 min of DBD-HVCP treatment or 10–60 kV for 10 min, depending on the plasma system. “Intense plasma exposure” refers to conditions that exceed the optimal range within a given experimental system, such as treatment times longer than 10 min in the same DBD-HVCP system, where decreases in surface hydrophobicity and reductions in actin and myosin heavy chain band intensities indicate excessive oxidation, denaturation, and aggregation or fragmentation. These numerical ranges should not be generalized across plasma systems because the effective treatment intensity also depends on discharge power and frequency, gas composition, electrode configuration, treatment distance, sample geometry, and matrix composition. The transition between these two regimes represents the dose-dependent “sweet spot” between desirable unfolding and deleterious aggregation [145].

From an application perspective, CP shows significant potential for improving the gelling properties of meat proteins, which has been particularly well documented in the case of surimi protein isolates [155,156,157] and myofibrillar proteins [144,158]. Nevertheless, improvements in gel formation should not be assumed to coincide automatically with enhanced emulsification or water retention, because individual functional properties may respond differently to the same oxidative and conformational modifications. Consequently, comprehensive evaluation should include protein solubility, aggregation, gel strength, rheological behaviour, emulsifying activity and stability, water-holding capacity, and relevant sensory attributes. Moderate relaxation of the protein structure also promotes increased adsorption at oil–water and air–water interfaces, but this structural change should be considered beneficial only when it is accompanied by measurable improvements in emulsifying activity, emulsion stability, foaming capacity, or foam stability [145,159]. Conversely, extensive unfolding or oxidation may reduce protein solubility or promote aggregation, thereby impairing interfacial functionality.

In addition to the direct modification of meat proteins, CP technology is used to modify other biopolymers, processes and macrostructures of food raw materials. However, plasma-induced molecular or structural changes should not automatically be interpreted as beneficial. Their practical relevance should be established by linking them to measurable technological, nutritional, sensory, microbiological, or toxicological outcomes.

Microstructure and macrostructure modification: The interaction of CP with meat matrices may modify their microstructure and surface morphology, including the development of surface irregularities or microporosity [59,78,160,161]. Under appropriately controlled treatment conditions, such structural modifications may influence moisture migration and other mass-transfer phenomena and, consequently, affect the physicochemical and functional properties of meat constituents [70,145,161]. The technological significance of these changes should be verified using quantitative indicators such as brine or marinade uptake, diffusion coefficients, curing-agent distribution, cooking loss, water-holding capacity, texture, and processing yield. Increased porosity should not be considered advantageous if it simultaneously promotes excessive moisture loss, oxidation, microbial penetration, or undesirable textural changes. Further studies are required to determine whether these effects can be consistently used to enhance the diffusion of brine, marinade, or curing components in different meat matrices.

Packaging materials: State-of-the-art CP treatment can modify the surface properties of polymeric packaging materials, creating opportunities for the development of functional packaging systems suitable for meat and meat products. Plasma pretreatment may improve surface wettability, coating adhesion, interfacial compatibility as demonstrated by changes in contact angle, surface energy, coating bond strength, or coating uniformity [162]. It may also modify selected mechanical and barrier properties, which should be evaluated using measurable parameters such as tensile strength, elongation at break, oxygen transmission rate, and water-vapour transmission rate. The magnitude and direction of these effects depend on the polymer type and treatment conditions [162]. Potential improvements must also be assessed against possible adverse outcomes, including polymer degradation, loss of mechanical integrity, migration of low-molecular-weight compounds, and the formation of oxidation products with toxicological or sensory relevance.

Freezing and thawing processes: CP has been investigated as an auxiliary treatment in freezing and thawing processes. Any proposed improvement in ice-crystal formation or distribution should be confirmed by direct measurements of crystal size and morphology, freezing rate, drip loss, water mobility, tissue microstructure, and textural properties. Therefore, CP should not be assumed to promote uniformly distributed ice crystals or reduce structural damage unless these outcomes have been experimentally demonstrated for the specific product and processing system. The integration of CP or PAW with freezing and thawing may contribute to microbial decontamination and the retention of proteinaceous material [70,91]. However, these effects should be quantified using microbial counts, reductions expressed in log CFU per appropriate product unit, thawing or drip loss, protein concentration in the exudate, protein solubility, and oxidation markers. A microbial reduction should also be considered together with possible changes in texture, color, flavor, and nutritional quality. Extraction and bioactivity: Compared with conventional thermal processing, non-thermal technologies may better preserve heat-sensitive nutrients and biologically active constituents by limiting thermally induced degradation. In meat matrices, cold plasma may additionally modify protein conformation and intermolecular interactions, thereby influencing the release, solubility, digestibility, and functional activity of selected components. Such structural modifications should be regarded as advantageous only if they produce measurable improvements, for example, increased extraction yield, protein solubility, in vitro digestibility, peptide bioaccessibility, or retention of biologically active compounds. These effects are dose- and matrix-dependent: mild treatment may improve the accessibility and functionality of certain compounds, whereas excessive exposure can promote protein oxidation, amino acid modification, pigment degradation, and lipid peroxidation. The nutritional implications of these reactions should be evaluated by measuring essential amino acid retention, protein digestibility, peptide profiles, and the bioaccessibility of relevant nutrients. Their sensory and toxicological implications should be assessed using volatile-compound profiles, sensory evaluation, lipid- and protein-oxidation products, and, where relevant, potentially hazardous reaction products. Consequently, CP processing requires product-specific optimization to balance possible improvements in constituent accessibility and functionality with the preservation of nutritional and oxidative stability [13,52,69].

Modern meat products are complex, multi-component matrices in which proteins, lipids, salts, curing agents, antioxidants, and other functional ingredients can influence the generation, transport, and reactivity of plasma-derived reactive oxygen and nitrogen species (RONS). At the molecular level, RONS may induce protein unfolding, oxidation, cross-linking, or fragmentation and may modify the activity of tissue and microbial enzymes associated with product quality and spoilage. These molecular alterations are not intrinsically beneficial or detrimental. Their relevance depends on their relationship with measurable product outcomes. Under optimized conditions, these effects can improve selected techno-functional properties of myofibrillar proteins, including emulsification and gel formation when accompanied by increased emulsifying activity, emulsion stability, gel strength, storage modulus, water-holding capacity, or processing yield. In contrast excessive oxidation and aggregation may reduce protein solubility and digestibility and impair functional performance as reflected by reduced extractability, poorer interfacial activity, increased cooking loss, or deterioration of gel texture [52,143,144,161].

Similarly, lipid oxidation and pigment modification should be evaluated in relation to quantitative oxidation markers, instrumental color, pigment stability, volatile compounds, and sensory acceptance, rather than being inferred solely from the presence of plasma-generated reactive species. Potential toxicological consequences should also be considered by monitoring the formation of relevant oxidation products or other processing-induced compounds. Therefore, CP treatment should be optimized separately for each meat matrix by linking the applied treatment conditions and resulting molecular modifications to microbiological safety, technological functionality, nutritional quality, sensory acceptance, and toxicological safety [52,143,144,161].

### 5.2. Inactivation of Enzymes and Undesirable Bioactive Compounds

CP-generated reactive oxygen and nitrogen species (RONS) may modify the structure and activity of endogenous and microbial enzymes present in meat and meat products. The proposed mechanisms include oxidation of susceptible amino acid residues, modification of sulfhydryl and aromatic groups, disruption or formation of disulfide bonds, and changes in the secondary and tertiary structures of enzyme proteins. These transformations may alter active-site geometry, substrate binding, and catalytic activity [69,163]. In meat and meat products, plasma-induced enzyme modification may affect proteolysis, lipid oxidation, pigment stability, texture, flavor development, and microbial spoilage. Nevertheless, the extent and technological significance of these effects must be established separately for each enzyme, product, and CP treatment system. Inactivation of oxidative or microbial enzymes may delay quality deterioration during storage, but this effect should be claimed only when a measured reduction in specific enzyme activity is accompanied by improved oxidative stability, color retention, flavor, texture, or microbial shelf life in the investigated meat product. Conversely, suppression of endogenous proteolytic enzymes may limit desirable post-mortem tenderization and flavor development. Therefore, the technological consequences of enzyme modification depend on the enzyme type, meat composition, processing stage, storage conditions, and CP treatment parameters. RONS may also modify undesirable biologically active compounds or microbial metabolites in meat products under specific experimental conditions. A decrease in the concentration of a parent compound should not automatically be interpreted as its complete degradation, detoxification, or an improvement in chemical safety. The identification and quantification of intermediate and final reaction products, together with appropriate toxicological or bioactivity assessments, are required before plasma treatment can be regarded as a safe method for removing undesirable compounds from meat products. Excessive plasma exposure may also generate secondary reaction products through protein oxidation, lipid peroxidation, or nitrosation. Consequently, enzyme inactivation and the degradation of undesirable compounds should be evaluated together with changes in protein functionality, oxidative stability, color, flavor, nutritional quality, and chemical safety [52,69,143,164].

### 5.3. Effects on Meat Color and Texture

#### 5.3.1. Color

Meat color is one of the most important quality characteristics directly affecting consumer acceptance because product freshness is often assessed based on surface appearance [165]. In red meat, color depends mainly on the relative proportions of metmyoglobin (met-Mb), deoxymyoglobin (deoxy-Mb), and oxymyoglobin (oxy-Mb), with met-Mb accumulation contributing to surface browning [166]. The met-Mb-reducing system plays an important role in maintaining the desired color, whereas lipid oxidation may further intensify undesirable changes in color and aroma, particularly in ground beef [166]. Therefore, plasma-induced color changes should be evaluated using the CIELAB parameters L*, a*, and b*, together with the total color difference, ΔE*. Whenever possible, these instrumental measurements should be complemented by quantification of myoglobin and its redox forms, particularly oxy-Mb and met-Mb, to distinguish visually detectable color changes from pigment oxidation.

The effect of CP on meat color depends on the plasma source, treatment conditions, and meat matrix. Cheng, Chen, and Sun [167] showed that plasma-activated solution (PAS) caused time-dependent color changes in met-Mb and oxy-Mb. Extended exposure of 0–10 min caused met-Mb to become green, whereas oxy-Mb changed from bright red to reddish-brown and subsequently to green. These changes were attributed to the overall redox environment generated in PAS rather than solely to the accumulation of nitrate, nitrite, and H_2_O_2_. Myoglobin color is governed by the ligand bound to heme iron and its oxidation state. Thus, nitrite-derived species may participate in nitrosylation-related reactions, whereas H_2_O_2_ and secondary oxidants, including •OH and ONOO^−^, may promote heme oxidation and oxidative modification of the globin moiety. Therefore, lower concentrations of reactive species may be insufficient to induce substantial color or structural changes, whereas higher PAS reactivity may shift the balance toward pigment oxidation and discoloration [168]. Accordingly, changes in L*, a*, b*, and ΔE* should be interpreted together with oxy-Mb and met-Mb levels rather than attributed to a single reactive species or color coordinate.

In chicken meat, DBD treatment significantly affected the color parameters, increasing L* and b* and decreasing a*, with argon providing the greatest color stability among the tested gases [78]. Plasma-activated acetic acid also reduced *Salmonella* Typhimurium counts and increased L*, with only minor changes in b* [169]. However, reporting individual CIELAB coordinates alone does not establish whether the overall color change is perceptible or whether it results from myoglobin oxidation. Future studies should therefore report ΔE* and, where relevant to the matrix, quantify total myoglobin, oxy-Mb, deoxy-Mb, and met-Mb.

#### 5.3.2. Texture

Based on the available evidence, the effects of CP on meat texture depend on the plasma source, treatment intensity, working gas, exposure time, and characteristics of the meat matrix. Consequently, the absence of adverse textural changes or an improvement in tenderness reported for a particular treatment should not be generalized to other plasma systems, muscles, species, or meat products. Under appropriately optimized conditions, CP may preserve the tenderness and textural stability of meat products without causing significant quality deterioration in specific meat matrices [59,88,91,92]. For example, in-package atmospheric DBD cold plasma did not significantly affect the tenderness of cooked chicken breast cubes during storage, while PAW used as a beef-thawing medium maintained physicochemical and sensory quality and limited structural changes in muscle proteins under the experimental conditions investigated [88,91]. These results demonstrate texture preservation in the respective products but do not establish a universal protective effect of CP on meat texture.

In some meat systems, CP may improve tenderness rather than merely preserve it. DBD-CP treatment of chicken breast reduced shear force and modified the structure of muscle fibres and connective tissue, with the strongest effects observed in argon-treated samples [95]. Similarly, high-voltage DBD-CP treatment of pork loin increased myofibrillar fragmentation and disrupted selected sarcomere structures, leading to improved tenderness, particularly at 70 kV [161]. However, these improvements were obtained for specific intact-muscle products and treatment conditions. They should not be interpreted as evidence that CP generally improves tenderness in all meat products. The textural response may differ according to the initial muscle structure, connective-tissue content, post-mortem state, proteolytic activity, treatment intensity, and subsequent storage conditions. The effect of CP on texture is closely related to changes in myofibrillar proteins and water retention. Mild or moderate treatment may induce partial protein unfolding and increase molecular flexibility, potentially improving protein interactions, emulsification, water-holding capacity, and gel formation [144,145,158,161]. These mechanisms are particularly relevant to comminuted, emulsified, and heat-induced gel meat products. In intact muscle, tenderness may additionally be influenced by sarcomere integrity, myofibrillar fragmentation, connective-tissue structure, endogenous proteolysis, and post-treatment storage. Therefore, changes in protein functionality observed in comminuted systems or isolated myofibrillar proteins should be distinguished from direct measurements of tenderness in intact meat. Results obtained for isolated myofibrillar proteins should not be directly extrapolated to the tenderness of intact muscle.

Excessive plasma exposure may promote protein oxidation, carbonyl formation, sulfhydryl loss, cross-linking, and aggregation, potentially reducing protein solubility and altering water retention or gel properties or textural characteristics [143,146,147,148]. During storage, these oxidation reactions may continue or interact with endogenous proteolysis, producing time-dependent and potentially opposing effects on texture. Proteolysis and limited structural disruption may contribute to tenderization, whereas extensive oxidation, aggregation, moisture loss, or connective-tissue modification may increase toughness or impair gel texture. Therefore, measurements performed immediately after CP treatment may not reflect the final textural response during refrigerated storage or after cooking.

Nevertheless, the final textural response is matrix-dependent. Recent research on refrigerated pork found no significant changes in tenderness after CAP treatment, despite increased water retention at longer exposure times [128]. This finding also indicates that improved water retention does not necessarily translate into measurable tenderization. Therefore, texture should be evaluated using shear force or texture-profile analysis together with water-holding capacity, cooking loss, protein oxidation, microstructure, and sensory assessment [78,128,161]. Claims of improved tenderness should consequently be restricted to the specific product, plasma system, processing conditions, storage period, and analytical method for which the effect was directly demonstrated.

### 5.4. Potential of Cold Plasma in Sodium-Reduction Strategies

CP is gaining increasing interest as an innovative technology supporting the development of sodium-reduced foods, although it does not directly remove sodium or reduce its concentration in the product [170]. Its potential contribution should be considered in terms of four distinct mechanisms: changes in brine diffusion, salt distribution, protein functionality, and perceived saltiness. CP-induced modifications of surface morphology and mass-transfer properties may influence brine penetration and sodium distribution within the product. However, improved diffusion or more uniform salt distribution does not necessarily indicate a reduction in total sodium content and should be confirmed by quantitative sodium analysis. Plasma treatment may also modify protein structure, hydration, and texture, potentially affecting sodium release from the food matrix and its accessibility during oral processing [105,170,171]. These effects should be distinguished from direct modulation of taste receptors, for which evidence in meat products remains limited. Any enhancement of perceived saltiness should therefore be verified by sensory evaluation and related to the measured sodium content, salt distribution, texture, and release kinetics. CP may additionally support the microbiological safety of meat products formulated with reduced NaCl concentrations. DBD-ACP treatment has been reported to inactivate pathogens in selected low-salt meat products [79,123]. Nevertheless, microbial inactivation, shelf-life extension, and sensory acceptance should be evaluated separately for each product, plasma system, sodium-reduction level, and storage condition. Thus, CP should be regarded as a complementary technology within sodium-reduction strategies rather than as a method that directly decreases sodium content.

### 5.5. Degradation and Mitigation of Polycyclic Aromatic Hydrocarbons

Cold plasma may reduce the formation of polycyclic aromatic hydrocarbons (PAHs) during high-temperature meat processing. In-package cold plasma pretreatment decreased PAH formation in charcoal-grilled beef steaks by 35–96%, with the strongest effect observed when grilling immediately followed plasma treatment [172]. The reduction in PAH8 content was associated with increased radical-scavenging activity of raw meat [172]. However, a decrease in the concentration of parent PAHs should not automatically be interpreted as their complete degradation, detoxification, or elimination of toxicological risk. In roasted beef patties, CP also degraded individual PAHs in standard solutions by 7.69–62.67% and reduced PAH8 levels in the product by 28.10–88.74%, depending on the treatment method and added oil or fat [173]. Results obtained in standard solutions should be distinguished from those obtained directly in the meat matrix because the composition of the product may influence plasma-induced reaction pathways and the resulting transformation products. This effect was associated with changes in fatty acid composition, including a lower proportion of PUFA and a higher proportion of MUFA. Nevertheless, reduced concentrations of the quantified parent PAHs do not exclude the formation of partially oxidized, oxygenated, nitrated, or otherwise modified derivatives that may retain or exhibit different toxicological properties.

Therefore, evaluation of CP-assisted PAH mitigation should include the identification and quantification of plasma-induced transformation products, assessment of the mass balance between parent compounds and their derivatives, and appropriate toxicity testing. Toxicological evaluation should address relevant endpoints such as cytotoxicity, genotoxicity, mutagenicity, and, where applicable, bioaccessibility. Until such evidence is available, CP treatment should be described as reducing the concentration or formation of selected PAHs under the investigated conditions rather than eliminating PAH-related toxicological risk. However, the identities and toxicological properties of plasma-generated PAH degradation products require further investigation before industrial implementation [173].

## 6. Indirect Treatment Strategies Using Plasma-Activated Water

The direct use of CP may be limited in some systems by undesirable quality effects, such as color loss, changes in surface topography resulting from plasma etching processes, and degradation of sensitive bioactive compounds [111]. Consequently, there is growing interest in indirect CP-treatment strategies, particularly plasma-activated water (PAW). PAW is produced through the transfer of plasma-generated reactive species from the gas phase into water, followed by their dissolution and subsequent aqueous-phase reactions. This process generates a mixture of relatively short- and long-lived reactive oxygen and nitrogen species and may alter water properties such as pH, oxidation–reduction potential, and electrical conductivity. The physicochemical composition and antimicrobial activity of PAW are not fixed properties but depend on the plasma source and configuration, working gas, activation time, electrical operating parameters, gas–liquid contact conditions, and the initial composition of the water. They may also change after activation depending on storage time, temperature, light exposure, and the type of storage container. Therefore, PAW should be characterized at the time of application, including, whenever possible, its pH, oxidation–reduction potential, electrical conductivity, and concentrations of relevant reactive species. In meat processing, PAW can be applied by spraying, immersion, thawing, or as a curing medium, enabling the separation of plasma generation from direct product treatment [174]. PAW is produced by exposing water to atmospheric-pressure plasma, resulting in the formation of reactive oxygen and nitrogen species. PAW has been investigated for surface disinfection and food decontamination [175]. In meat systems, PAW applied by spraying, immersion, or as a thawing or curing medium may reduce microbial contamination. However, antimicrobial effectiveness should be interpreted in relation to the physicochemical properties of the PAW used, the target microorganism, treatment conditions, organic load, and characteristics of the meat matrix. Its effects on color, oxidation, texture, and sensory quality are likewise dependent on the application method, PAW composition, treatment intensity, and meat matrix. Accordingly, findings obtained with one PAW-generation system or storage protocol should not be generalized to PAW produced or applied under different conditions. The shelf-life of PAW is primarily determined by the storage stability of its reactive species and physicochemical properties. Therefore, PAW stability should be evaluated by monitoring pH, oxidation–reduction potential (ORP), electrical conductivity, and the concentrations of H_2_O_2_, NO_2_^−^, and NO_3_^−^ at defined intervals throughout storage. Although some parameters such as pH and nitrate concentration, may remain relatively stable during refrigerated conditions, nitrite and hydrogen peroxide concentrations may change progressively, potentially altering the antimicrobial activity of PAW. Zhang et al. [49] reported that during storage at 4 °C for 30 days, pH, ORP, and conductivity remained relatively stable, while the contents of NO_3_^−^, NO_2_^−^, and H_2_O_2_ changes significantly after stored for 5 days. These changes were attributed to the gradual transformation of reactive species under acidic conditions, even at low storage temperatures, and may affect the physicochemical and antimicrobial stability of PAW. However, these findings apply to the specific PAW formulation, plasma-generation system, water composition, storage temperature, and analytical conditions investigated and should not be generalized to all PAW systems. PAW samples stored under refrigeration may retain selected long-lived reactive species, such as H_2_O_2_ and NO_2_^−^, more effectively than samples stored at room temperature [176]. However, this effect is not universal because the stability of these species depends on the plasma source, working gas, activation parameters, initial water composition, and storage conditions. Antioxidant activity and radical-related responses have been detected at low levels for up to 60 days in specific PAW formulations, but these findings should not be generalized to PAW generated using different activation procedures or water compositions.

Studies have reported reductions in selected foodborne pathogens following treatment with PAW or other plasma-activated liquids. Sammanee et al. [95] reported a reduction in *Campylobacter* spp. by 1.03 ± 0.99 log CFU/g in pork following PAW treatment. Kang et al. [169] observed a reduction in *Salmonella* Typhimurium by 1.23 log CFU/cm^2^ on chicken meat after a 10-min treatment with plasma-activated acetic acid. These results should be interpreted separately rather than directly compared because the studies differed in meat matrix, target microorganism, plasma-activated treatment medium, treatment conditions, sampling procedure, and reporting unit. In particular, reductions expressed as log CFU/g and log CFU/cm^2^ are not directly comparable because they refer to microbial contamination relative to product mass and surface area, respectively. Therefore, these findings demonstrate treatment- and matrix-specific antimicrobial effects but do not establish a common level of efficacy for PAW or other plasma-activated liquids across meat products.

Integrating microbiomic and metabolomic analyses of volatile compounds in high-fat poultry model systems is of particular importance. A study by Ju et al. [94] using advanced tools such as 16S rRNA sequencing and HS-SPME-GC-MS showed that *Staphylococcus saprophyticus* is the dominant microorganism responsible for spoilage of duck fillets during refrigerated storage. The use of PAW not only effectively reduced the number of these bacteria but also reduced lipid and protein oxidation and mitigated unfavorable changes in the volatile compound profile, which translated into extended shelf life at 4 °C. Therefore, PAW represents an optimal, non-thermal preservation strategy that simultaneously maintains the chemical and sensory quality of delicate poultry products [94]. These findings indicate that PAW may be a promising non-thermal preservation strategy for selected poultry products under optimized conditions. However, the results are specific to the investigated duck-fillet model, PAW-generation procedure, treatment conditions, and storage temperature and should not be generalized to other poultry products or processing systems. Additional validation is required to establish the reproducibility of these effects, their influence on sensory acceptance, the toxicological safety of PAW-induced reaction products, and the feasibility of industrial-scale implementation.

## 7. Adverse Effects of Cold Plasma and Mitigation Strategies

Despite its potential technological applicability, the use of CP in high-fat meat products may accelerate oxidative reactions [80]. Certain technological configurations, particularly in-package DBD-CP treatment, may alter the volatile-compound profile, as reflected by increased concentrations of aldehydes, alcohols, and esters [88,177]. These changes may result from lipid and protein oxidation and should not automatically be interpreted as technologically or sensorially beneficial. Therefore, treatment conditions should be optimized separately for each meat matrix to achieve sufficient microbial inactivation while maintaining lipid, protein, and pigment stability and limiting undesirable changes in volatile compounds. The evaluation of CP-treated high-fat meat products should combine microbial counts with appropriate indicators of lipid and protein oxidation, instrumental color and pigment measurements, volatile-compound profiling, and sensory assessment. A summary of the main side effects of CP/ACP/PAW use and factors that exacerbate their occurrence is provided in Table 5.

ROS generated during ACP processing can initiate lipid oxidation through reactions with unsaturated fatty acids, leading to the formation of secondary oxidation products (aldehydes, alcohols, and ketones) characterized by low odor thresholds and directly responsible for the development of undesirable flavor notes [120]. Analyses of the volatile compound profile (GC-IMS and GC-MS) indicate that compounds such as hexanal, nonanal, hexyl acetate, benzaldehyde, (*E*)-2-alkenals, and 2-hexanol are formed mainly as a result of lipid autoxidation and degradation of their hydroperoxides, imparting rancid, sharp, or bitter almond notes to the meat [120,122,177]. The intensity of these changes is usually positively correlated with the extension of processing time and the increase in process voltage [122]. In the case of dried beef, the use of DBD ACP with atmospheric air resulted in a slight decrease in flavor scores and an increase in the intensity of undesirable odor with increasing processing intensity [126]. Comparison of undesirable changes occurring in meat and fish products after the use of CP allows for the identification of the main areas of quality risk, especially in lipid-rich matrices. A summary of these changes is presented in Table 6.

Limiting these unfavorable changes requires the implementation of appropriate mitigation strategies. The basic solution is precise control and optimization of process parameters (elimination of oxygen from the process atmosphere and the use of inert gases, e.g., pure argon, reduction in voltage/power, shortening of exposure time, reduction in fat content in the raw material) [80,120,179]. The second highly effective strategy involves the use of antioxidants, including naturally occurring compounds such as vitamin E, which comprises tocopherols and tocotrienols. Owing to its ability to scavenge reactive oxygen species and free radicals, vitamin E can effectively mitigate CP-induced lipid oxidation, thereby improving the oxidative stability of lipid-rich products [180,181,182,183]. However, caution should be exercised when dosing, as at excessively high concentrations some forms of tocopherols may exhibit undesirable prooxidant effects [184]. However, the protective effect of vitamin E is concentration- and matrix-dependent. In meat systems, α-tocopherol has been shown to improve oxidative stability at levels such as 100–200 mg/kg meat in chicken patties and 200 ppm in beef sausages [185,186]. However, lipid model systems indicate that α-tocopherol may lose efficacy or even promote radical formation at excessive concentrations; for example, concentrations up to 0.35 mg/g fatty acid or triglyceride inhibited lipid radical adduct formation, whereas higher concentrations intensified adduct formation [160]. Therefore, no universal pro-oxidant threshold can currently be defined for meat matrices, and vitamin E supplementation in CP-treated high-fat foods should be optimized for each product using lipid oxidation markers such as TBARS, peroxide value, hexanal formation, and sensory quality.

The third key path is to replace direct treatment with indirect methods, such as the application of PAW, as described above [90,91,94]. Reducing the undesirable effects of CP requires a comprehensive approach, including the selection of process parameters, gas atmosphere, exposure time, packaging type, and storage conditions. Proposed directions for designing a “quality-safe” CP process are presented in Table 7.

## 8. Chromatographic and Electronic-Nose Analysis of Volatile Compounds in Plasma-Treated Meat

Although CP an effectively reduce microbial contamination on meat surfaces, its efficacy and chemical effects depend on the treatment conditions and the properties of the meat matrix. Plasma-generated RONS exhibit strong electrophilic properties and may initiate the oxidation of food components, particularly PUFA-containing lipids and proteins [86]. Because lipid and protein oxidation contribute to the formation and degradation of aroma compounds, these reactions may alter the volatile and, consequently, the flavor profile of meat products [187]. Secondary lipid oxidation leads to the formation of low-molecular-weight VOCs, which may substantially deteriorate the flavor profile of food by generating rancid, grassy, and paint-like notes, thereby shortening its sensory shelf life. Understanding and controlling these phenomena requires advanced, sensitive, and rapid analytical systems. Classical oxidation indicators, such as peroxide value (PV) and the TBARS test, provide useful global measures of primary and secondary lipid oxidation, respectively, but offer limited, non-specific information about individual oxidation products [188]. Therefore, these indicators should be complemented by targeted and untargeted analyses of both volatile and non-volatile oxidation products. In particular, profiling VOCs after plasma exposure, together with the identification of non-volatile oxidation markers, enables a more comprehensive assessment of oxidative changes and supports a reliable evaluation of the quality and safety implications of implementing this technology in the meat sector [178,189].

Meat aroma depends on the relative abundance of numerous groups of volatile compounds, including aldehydes, ketones, alcohols, esters, acids, hydrocarbons, and sulfur- and nitrogen-containing compounds. The qualitative and quantitative profiles of these compounds are influenced by animal species, lipid oxidation, amino acid degradation, and other protein-derived reactions and may therefore provide information about meat quality, freshness, and storage-related deterioration [120,190].

Among these compounds, aldehydes are particularly important. Hexanal is widely recognized as a marker of linoleic acid oxidation and an indicator of oxidative deterioration in meat. Other aldehydes, including nonanal, octanal, and pentanal, as well as selected ketones and alcohols, may also change in relative abundance depending on the product composition and processing, packaging, and storage conditions. Analysis of these compounds provides information on the current oxidative status of the product and may help monitor the progression of quality changes during storage [189].

In plasma-treated meat, volatile analysis can serve a dual purpose: detecting oxidation induced by plasma treatment and distinguishing these effects from changes associated with natural ageing and microbial spoilage. Accordingly, a combination of complementary analytical techniques, including volatile profiling and global or targeted oxidation measurements, is preferable to reliance on a single oxidation marker. Modern food analytics is based on a complementary instrumental triad:

(1) HS-SPME-GC-MS (Headspace Solid-Phase Microextraction coupled with Gas Chromatography–Mass Spectrometry) is one of the most important methods for analyzing volatile compounds in food. It involves the extraction of analytes from the sample headspace using a sorbent-coated fibre, followed by their chromatographic separation and identification by mass spectrometry. HS-SPME is a solvent-free and relatively simple technique that is particularly useful for analyzing complex food matrices [191]. It is commonly used for the identification of VOCs in studies evaluating the effects of CP treatment on food [192]. However, the obtained volatile profile and the comparability of results depend on several methodological factors, including the fibre coating, extraction temperature, equilibration and extraction times, internal standards, chromatographic conditions, and compound-identification criteria. Therefore, these parameters should be standardized and reported in detail.

GC-IMS (Gas Chromatography–Ion Mobility Spectrometry) provides rapid and sensitive profiling of volatile compounds and is particularly useful for sample discrimination and monitoring changes in volatile fingerprints. However, unequivocal compound identification may be limited when authentic standards and complementary mass-spectrometric data are unavailable. Therefore, GC-IMS is especially valuable for comparative profiling, whereas the identities of relevant compounds may require confirmation using complementary analytical techniques.

Electronic-nose systems are useful for rapid screening, classification, and monitoring of changes in the overall volatile profile of meat products. Nevertheless, these systems produce pattern-based responses and do not directly identify or quantify individual volatile compounds. Consequently, electronic-nose measurements should be complemented by chromatographic and mass-spectrometric techniques when the identification and quantification of specific VOCs are required. When used to assess the effects of CPs, it is characterized by unrivaled resolving power, precise structural assignment (aldehyde isomers), and extensive spectrometric databases. HS-SPME-GC-MS can detect both freshness markers and characteristic products of lipid oxidation and protein degradation. This allows for a detailed assessment of whether CP exposure increases the concentration of compounds responsible for undesirable odor notes and whether this effect intensifies during storage. In research practice, HS-SPME-GC-MS is used, among others, to monitor: changes in the concentration of hexanal, nonanal, pentanal, and other aldehydes; increases in the content of ketones and alcohols as secondary oxidation products; the appearance of sulfur compounds associated with protein degradation; and differences between control and plasma-treated samples during different periods of refrigerated storage. However, the challenge remains the phenomenon of competitive adsorption on the fiber (main matrix components can block active sites for trace oxidation markers); long analysis time (>40 min); the lack of direct online implementation in an industrial process; the need for careful optimization of extraction conditions; and the fact that interpretation of results requires experience and appropriate mass spectral libraries [183].

(2) Ion mobility spectrometry coupled to fast gas chromatography (GC-IMS) represents a modern breakthrough in monitoring volatile lipid oxidation products [193]. The IMS detector is characterized by extraordinary sensitivity for compounds with high proton affinity, such as aldehydes, ketones, and alcohols—key markers of secondary lipid oxidation. The limits of detection (LOD) for these compounds in GC-IMS reach ppb (μg/kg) and even ppt levels, allowing detection of the initial stages of rancidity much earlier than classical sensory or chromatographic methods [183]. Furthermore, the analysis takes only 10–15 min. and does not require the use of solvents or expensive consumables. This method is gaining importance in food analysis due to its high sensitivity, short response time, and good suitability for comparative and classification studies [194]. In studies of CP-treated meat, GC-IMS can be used as a tool for quickly differentiating samples in terms of qualitative changes. It allows for the observation of changes in entire odor patterns without the need for immediate, unambiguous identification of all components. This is particularly useful when examining a series of samples that differ in plasma exposure time, working gas type, or storage length [194].

(3) The electronic nose is a biomimetic instrumental platform that generates a digital fingerprint of a sample’s odor without separating it into individual chemical entities. The electronic nose serves as an ideal tool for immediate qualitative assessment (screening). It allows for the classification of samples by degree of sensory degradation immediately after the CP process, acting as an early warning system on packaging or production lines. Unlike GC-MS, which identifies individual compounds, the e-nose provides information on the sample’s global aroma pattern, thus functionally approaching sensory evaluation, but in an objective, rapid, and repeatable manner. The advantage of the e-nose is the ability to very quickly detect differences between samples, even when changes in the concentrations of individual compounds are still relatively small but the overall odor profile has already been modified. In practice, this means that the e-nose can be used as an early warning system against the development of unfavorable oxidative and sensory changes after plasma treatment [195]. The primary limitation of the electronic nose is the lack of direct chemical identification of the compounds responsible for the observed signal change. Sensor response depends on the overall mixture standard, humidity, temperature, and stability of the measurement system. Therefore, the e-nose does not replace GC-MS or GC-IMS, but serves as a valuable complement. A complementary strategy is the use of humidity-tolerant or hydrophobic sensor coatings. Wang and Zhou [196] identified surface engineering, physical isolation, modulation of operating parameters, humidity compensation, and novel sensing materials as major anti-humidity strategies for chemiresistive gas sensors. In addition, Oliveira et al. [197] demonstrated that hybrid gel sensors based on a hydrophobic PDMS matrix reduced the effect of humidity on sensing performance compared with hydrophilic gelatin-based systems under 50% RH conditions. For meat applications, such coatings could help stabilize sensor responses in humid headspace environments. Finally, humidity effects can also be addressed computationally. Cai et al. [198] proposed a humidity compensation approach for e-nose data, while Damdam et al. [199] integrated temperature/humidity sensors into an IoT-enabled e-nose system for beef spoilage monitoring. Therefore, reliable e-nose operation in humid meat matrices should combine controlled headspace dehumidification, humidity-resistant sensor coatings, and humidity-compensated data processing. A comparison of the physicochemical and application parameters of the three platforms discussed in relation to the analysis of secondary products of CP-induced lipid oxidation is presented in Table 8 below.

In studies on meat quality after CP treatment, an integrated approach seems most rational. Given the compositional complexity of most food matrices, data fusion is a popular analytical strategy, which combines data from complementary instrumental techniques and can provide more information and generate more reliable conclusions than a single technique [204,205]. The highest level of analytical precision in monitoring the effect of CP on fat quality is achieved by integrating data from multiple measurement platforms and subjecting them to advanced chemometric analyses [201]. This analytical sequence optimizes both study time and interpretation depth. E-nose can be used to initially identify samples with the greatest aromatic differences. GC-IMS then enables a more detailed comparison of volatile metabolite patterns and chemometric analysis. Finally, HS-SPME-GC-MS allows for attributing observed differences to specific chemical compounds and linking them to mechanisms of lipid oxidation or protein degradation [194]. In CP research, this approach is particularly valuable because qualitative changes are usually multifactorial and depend simultaneously on processing parameters and storage conditions. Using only one technique can lead to oversimplification of interpretation.

## 9. Electrophoretic, Conformational, and Molecular Methods for Evaluating Plasma-Induced Changes

Meat, as a highly organized and hydrated multiphase system with a dominant protein fraction (mainly myofibrillar, sarcoplasmic, and connective tissue proteins) and lipid [206], undergoes multidirectional physicochemical changes under the influence of CAP. For food analysts and meat technologists, it is crucial not only to record these changes, but also to correlate them quantitatively and qualitatively with the functional properties of the raw material, such as water-holding capacity (WHC), color, texture, and sensory profile [207]. Traditional methods for assessing the degree of oxidation (e.g., determination of carbonyl groups using the DNPH method or the TBARS index for lipids) provide only summary information on the state of the matrix, often masking subtle, local structural changes.

### 9.1. Electrophoretic Assessment of Protein Aggregation and Fragmentation

Electrophoretic methods enable visualization of molecular-weight profiles, changes in protein-band intensity, aggregation, cross-linking, and fragmentation, as well as assessment of the purity of individual protein fractions [150,196].

Sodium dodecyl sulfate-polyacrylamide gel electrophoresis (SDS-PAGE) remains a fundamental method for assessing changes in the integrity, polymerization, aggregation, and fragmentation of muscle proteins after CAP treatment. However, SDS-PAGE provides information primarily on changes in protein migration patterns and does not directly identify specific oxidation, nitration, or other chemical modifications or determine their locations within protein sequences. Therefore, electrophoretic observations should be complemented by mass-spectrometric proteomic methods or targeted chemical analyses when identification and localization of specific protein modifications are required.

Plasma-generated reactive species can induce oxidative modifications of proteins at or near the treated meat surface. Short-lived species are expected to react locally because of their high reactivity and limited lifetime. Longer-lived species, including H_2_O_2_ and O_3_, may persist sufficiently to undergo transport and secondary reactions within surface moisture or the superficial tissue layer. However, their penetration depth in meat has not been adequately quantified and should not be inferred solely from chemical changes detected in bulk samples. Therefore, CAP-induced protein modification in meat should be described as a combination of surface reactions, transport of relatively persistent species, and secondary oxidative chemistry rather than as uniform penetration of RONS throughout the meat matrix. Cysteine (Cys), methionine (Met), tryptophan (Trp), and lysine (Lys) residues are among those particularly susceptible to modification [197]. Although these reactions may produce characteristic changes in SDS-PAGE patterns, the underlying chemical mechanisms cannot be established solely from the electrophoretic image.:

These changes may be reflected in the electrophoretic pattern in two principal ways:

(1) Oxidative cross-linking: Oxidation of thiol (-SH) groups in cysteine residues may promote the formation of covalent disulfide bonds (-S-S-), contributing to protein cross-linking and aggregation. Depending on the oxidative conditions generated during CAP treatment, cysteine residues may also undergo further oxidation to sulfenic (-SOH), sulfinic (-SO_2_H), or sulfonic (-SO_3_H) derivatives. Other oxidative cross-links, including dityrosine species, may also be formed in oxidatively stressed proteins [208,209]. Reactions between protein amino groups and carbonyl compounds generated through protein or lipid oxidation may additionally contribute to the formation of high-molecular-weight products. In SDS-PAGE, protein aggregation may be indicated by a decrease in the intensity of monomeric protein bands, particularly the myosin heavy chain, accompanied by the appearance or accumulation of high-molecular-weight material near the interface between the stacking and resolving gels. Nevertheless, these electrophoretic changes do not, by themselves, confirm the formation of specific chemical bonds [208].

(2) Oxidative fragmentation: Radical-mediated reactions may cause cleavage of the peptide backbone and generate lower-molecular-weight protein fragments or peptides. In SDS-PAGE, this process may be reflected by the appearance of additional low-molecular-weight bands, reduced intensity of the original protein bands, or increased smearing. However, electrophoretic patterns alone cannot identify cleavage sites or unequivocally establish the chemical pathway responsible for fragmentation. Identification and localization of oxidation products require more selective approaches, particularly LC-MS/MS-based methods [209].

To support the interpretation of the bonds contributing to protein aggregation, samples can be analyzed under both reducing and nonreducing conditions:

(3) Electrophoresis under reducing conditions: Reducing agents, such as dithiothreitol or β-mercaptoethanol, disrupt disulfide bonds. Dissociation of high-molecular-weight aggregates into monomeric forms after reduction supports the involvement of disulfide-mediated cross-linking but does not exclude the simultaneous presence of other covalent modifications.

(4) Electrophoresis under nonreducing conditions: The omission of reducing agents preserves disulfide-linked aggregates. Accordingly, comparison of reducing and nonreducing electrophoretic patterns can help assess the contribution of disulfide bonds to aggregation. The persistence of high-molecular-weight material under reducing conditions may suggest the presence of stable nondisulfide covalent cross-links. However, confirmation of specific structures, including dityrosine cross-links, as well as identification of oxidation, nitration, and other modification sites, requires complementary targeted chemical analyses or mass-spectrometric methods [198,208,209].

### 9.2. Conformational and Spectroscopic Methods

Subtle changes in protein spatial architecture, occurring before or simultaneously with aggregation, require sensitive spectroscopic and polarimetric techniques. FTIR spectroscopy, steady-state fluorescence spectroscopy, and circular dichroism provide complementary information on different levels of protein organization. FTIR and far-UV CD are primarily used to assess secondary-structure changes, whereas intrinsic fluorescence spectroscopy provides information on the local environment of aromatic residues and changes in tertiary structure. However, in complex meat matrices, the interpretation of these measurements may be affected by spectral overlap, sample heterogeneity, water interference, light scattering, and the applied data-processing procedures. Therefore, conclusions regarding protein conformational changes should preferably be supported by more than one complementary technique and based on standardized sample-preparation, measurement, and data-analysis protocols [197,199].

#### 9.2.1. FTIR Spectroscopy (Fourier Transform Infrared)

Fourier transform infrared spectroscopy allows monitoring of changes in the secondary structure of proteins [210,211]. A key interpretative approach involves analysis of the Amide I band at 1600–1700 cm^−1^, which is mainly associated with C=O stretching vibrations of the peptide backbone, with minor contributions from C–N stretching and N–H bending vibrations. Because the raw Amide I band is usually a broad and asymmetric envelope, second-derivative analysis, Fourier self-deconvolution, and curve fitting using Gaussian or Lorentzian components are commonly applied to resolve overlapping structural contributions. However, the estimated proportions of individual secondary-structure components may depend on baseline correction, smoothing, deconvolution parameters, the number and position of fitted components, and the selected fitting model. Consequently, identical preprocessing and curve-fitting procedures should be used for all samples, and the analytical parameters should be reported in sufficient detail.

When FTIR spectroscopy is applied to whole meat, meat batters, or other highly hydrated muscle-protein matrices, the contribution of water to the infrared spectrum must be carefully considered. The H–O–H bending band of water occurs near 1645 cm^−1^ and may interfere with the Amide I region used to estimate protein secondary structure. Moreover, differences in water content and mobility can reflect actual changes in protein hydration, hydrogen bonding, and conformation rather than spectral interference alone. Studies on pork meat batters, bovine muscle tissue, and processed pork have demonstrated close relationships between water distribution, protein hydration, and secondary-structure parameters determined by FTIR [212,213,214].

Sample heterogeneity, including variations in fat, connective tissue, moisture, and protein distribution, may additionally affect spectrum reproducibility and complicate comparisons between treatments. Therefore, comparisons between CP-treated and control samples should be based on identical sample-preparation, hydration, temperature, spectral-acquisition, and preprocessing conditions. For hydrated samples, an appropriate water or background spectrum should be subtracted before second-derivative analysis, Fourier self-deconvolution, or curve fitting of the Amide I band. Controlled dehydration or freeze-drying may reduce water interference, but these procedures should be applied cautiously because water removal may alter hydrogen bonding, protein aggregation, and secondary structure. Consequently, FTIR-derived changes in α-helix, β-sheet, β-turn, and random-coil content should be interpreted together with measurements of water distribution, water-holding capacity, or moisture content [212,213,214].

Thus, CAP-induced changes in myofibrillar protein structure, such as decreases in α-helical structures at 1650–1658 cm^−1^ and increases in β-sheet structures at 1615–1638 cm^−1^ or random-coil structures at 1640–1648 cm^−1^, should be interpreted only when water interference has been minimized and the same acquisition and preprocessing protocol has been applied across all samples [197]. Where possible, the interpretation of these changes should be corroborated using a complementary conformational method, such as CD or fluorescence spectroscopy.

#### 9.2.2. Steady-State Fluorescence Spectroscopy

This method is based on the analysis of intrinsic protein fluorescence, which originates primarily from tryptophan residues. At an excitation wavelength of λ_ex_ = 295 nm, the contribution of tyrosine is minimized, and emission is typically recorded within the range of 300–400 nm. The wavelength and intensity of tryptophan fluorescence are strongly affected by the polarity and accessibility of its local environment, making this technique useful for monitoring changes in protein tertiary structure [215]. In the native protein structure, many Trp residues are located within relatively hydrophobic environments. RONS-induced disruption of interactions stabilizing the tertiary structure may expose these residues to a more polar solvent environment, producing measurable spectral changes. These changes may include [215]:

- Bathochromic or red shift: the maximum emission wavelength, λ_max_, shifts toward longer wavelengths, for example, from approximately 330 nm to above 340 nm, which may indicate increased exposure of Trp residues to a polar environment.
- Fluorescence quenching: emission intensity decreases because of collisional or static quenching, conformational changes, solvent exposure, energy transfer, or chemical modification of Trp residues, including the formation of oxidation products such as kynurenine and *N*-formylkynurenine

Nevertheless, changes in fluorescence intensity and λ_max_ are not determined exclusively by protein conformation. In heterogeneous meat matrices, the results may also be affected by turbidity, light scattering, inner-filter effects, protein concentration, pH, temperature, lipid-derived oxidation products, and interactions with other absorbing or fluorescent compounds. Light scattering can be particularly important in complex, particulate, or aggregated samples and may require appropriate background correction before the fluorescence signal is interpreted [215,216].

Plasma-induced chemical oxidation of Trp may also decrease fluorescence independently of conformational unfolding. Accordingly, fluorescence spectra should be corrected for background signals and inner-filter effects where relevant, and measurements should be conducted using consistent sample concentration, optical geometry, temperature, pH, and spectral-processing procedures. Fluorescence changes should therefore be interpreted as indicators of alterations in the local environment of aromatic residues rather than as direct and exclusive evidence of a specific tertiary-structure transition [215,216]. Where possible, the results should be corroborated using complementary methods such as FTIR or circular dichroism spectroscopy, because intrinsic fluorescence and CD provide information about different levels of protein structure [217].

#### 9.2.3. Circular Dichroism (CD)

CD is mainly used to assess changes in the secondary structure of myofibrillar proteins following PAW/CP treatment, particularly changes in the proportion of α-helix, β-sheet, β-turn, and random structures. The most frequently observed effect is partial loss of α-helical structure, increase in β- or random coil structures, and these changes are associated with protein oxidation, aggregation, and improvement or deterioration of the gelling properties of meat [207,218,219]. CD provides a rapid assessment of protein folding and secondary-structure composition, although it does not provide residue-specific structural information [220].

Far-UV circular dichroism (190–260 nm) provides a complementary, highly precise method for validating secondary structure in dispersive systems. α-helical structures generate two characteristic minima at 208–222 nm, while the β-pleated structure exhibits a single minimum around 218 nm. Molar ellipticity tracking allows for quantification of the plasma dose-induced conformational dynamics in real time.

However, quantitative interpretation of CD spectra requires accurate determination of protein concentration, optical path length, and mean residue ellipticity. Measurements of complex meat-protein dispersions may be affected by sample turbidity, light scattering, aggregation, particulate matter, and absorption by non-protein components. These effects are particularly relevant at lower wavelengths and may distort spectral shape or prevent reliable structural deconvolution. Therefore, CD analysis should preferably be performed using sufficiently clarified and homogeneous protein dispersions under standardized pH, ionic-strength, temperature, protein-concentration, and path-length conditions. The selected baseline correction, smoothing, and spectral-deconvolution method should also be reported. Changes in CD spectra should not be interpreted independently when substantial aggregation or scattering is present and should be corroborated using complementary techniques such as FTIR or fluorescence spectroscopy [220,221].

Overall, the three techniques should be regarded as complementary rather than interchangeable. FTIR provides information on vibrational signatures associated predominantly with secondary structure and protein hydration, intrinsic fluorescence probes the local environment of aromatic residues and tertiary-structure changes, and CD characterizes secondary structure in sufficiently homogeneous protein systems. Agreement among these methods strengthens the interpretation of CP-induced conformational changes, whereas divergent results should be evaluated in relation to matrix heterogeneity, water content, aggregation, spectral interference, and differences in data processing [217,221].

### 9.3. Molecular and Microbiome-Based Methods

Molecular and biosensor-based methods can be used to assess microbial and meat-cell nucleic acids, thereby providing information relevant to plasma-induced damage mechanisms [222]. However, the detection of microbial DNA does not necessarily demonstrate cell viability because DNA may persist after membrane damage or cell death. Therefore, DNA detection alone should not be used to determine microbial survival, biocidal efficacy, or the penetration depth of plasma effects.

Traditional culture-based methods may underestimate microbial viability following cold plasma treatment because the absence of colony formation does not necessarily demonstrate complete bacterial inactivation. Under adverse processing and preservation conditions, foodborne bacteria may become sublethally injured or enter a viable but non-culturable (VBNC) state. Such cells do not form visible colonies on routine culture media but may retain metabolic activity or membrane integrity, potentially leading to an underestimation of viable bacterial populations [223,224].

Conversely, culture-independent molecular methods may overestimate the number of viable microorganisms by detecting DNA from dead or irreversibly damaged cells. Accordingly, conclusions regarding microbial survival and food safety should not rely exclusively on either culture-dependent or DNA-based methods.

A more reliable assessment can be obtained by combining culture-based enumeration with viability PCR and sequencing-based approaches. Viability PCR uses membrane-impermeable DNA-intercalating dyes, such as propidium monoazide (PMA) or ethidium monoazide (EMA), to reduce the amplification of DNA from cells with compromised membranes. However, its results depend on membrane integrity, dye penetration, matrix composition, and treatment conditions and should not be interpreted as an absolute measure of viability. Where appropriate, measurements of metabolic activity or cell-resuscitation assays may further support the differentiation of culturable, VBNC, injured, and dead cells.

Metataxonomic analysis based on 16S rRNA gene amplicon sequencing can complement classical microbiology by characterizing changes in bacterial-community composition. The 16S rRNA gene contains conserved and hypervariable regions, and the V3–V4 region is commonly used because it generates an amplicon of approximately 460 bp suitable for bacterial-community analysis [225]. High-throughput sequencing has demonstrated that meat and meat-processing environments contain complex microbial communities, including spoilage-associated genera such as *Pseudomonas*, *Brochothrix*, *Psychrobacter*, and *Acinetobacter*, as well as lactic acid bacteria [226].

Nevertheless, conventional 16S rRNA gene amplicon sequencing detects bacterial DNA regardless of whether it originates from viable, injured, VBNC, or dead cells. Moreover, it generally provides relative rather than absolute abundance data, and its results may be influenced by DNA extraction efficiency, primer specificity, amplification bias, sequencing depth, and differences in 16S rRNA gene copy number among bacterial taxa. Consequently, an apparent increase in the relative abundance of a taxon does not necessarily indicate an increase in its absolute cell number. Amplicon sequencing can therefore characterize changes in bacterial-community composition but does not directly quantify viable pathogens, demonstrate their metabolic activity, or establish microbial safety.

Biodiversity analysis provides insight into ecological changes occurring within the meat microbiome [227]. α-Diversity, assessed using indices such as Shannon, Simpson, and Chao1, describes different aspects of bacterial diversity within an individual sample. CAP treatment may alter or reduce α-diversity, but such changes should not automatically be interpreted as the elimination of sensitive species. Lower diversity may reflect changes in relative abundance, reduced taxon detectability, sequencing depth, DNA extraction efficiency, or persistence of DNA from non-viable cells.

β-Diversity, commonly visualized using principal coordinates analysis (PCoA) based on weighted or unweighted UniFrac distances, describes differences in bacterial-community composition or phylogenetic structure among samples exposed to different plasma doses. Although t-distributed stochastic neighbor embedding (t-SNE) may also be used for dimensionality reduction, it does not represent phylogenetic distances in the same manner as UniFrac-based PCoA and should be interpreted separately.

A CAP-induced reduction in microbial diversity should not automatically be interpreted as improved food safety. Meat spoilage is driven by ecological succession and changes in community composition rather than by total microbial counts alone. Studies on refrigerated beef have shown that spoilage-associated microbiota change during storage and depend on packaging conditions, with different bacterial taxa becoming dominant under different atmospheres [16]. Plasma treatment should therefore be evaluated in terms of both α-diversity and β-diversity, including the possible relative enrichment of stress-tolerant or initially low-abundance taxa. However, neither α-diversity nor β-diversity provides direct information on the absolute abundance, viability, or infective potential of foodborne pathogens.

This ecological interpretation is particularly relevant because *Pseudomonas* spp. are common in meat and meat-processing environments and may interact with foodborne pathogens in biofilms, potentially affecting pathogen persistence and antimicrobial tolerance [228]. Conversely, the elimination of sensitive spoilage bacteria may create ecological opportunities for more resistant populations. Community-level sequencing should therefore be complemented by pathogen-specific culture-based enumeration, quantitative molecular assays, and storage validation under relevant packaging and temperature conditions before conclusions regarding microbial safety are drawn.

When microbial viability information is required, DNA-based metataxonomic analysis should be complemented with matrix-validated viability-sensitive approaches, such as PMA-assisted PCR or 16S rRNA gene sequencing and RNA-based assays, together with culture-based methods and pathogen-specific enumeration [229,230,231]. RNA-based approaches may provide additional information on cellular activity, but RNA detection alone should not be regarded as definitive proof of viability without appropriate validation.

The efficacy of PMA-assisted analysis should be validated separately for different meat matrices, including ground meat and intact muscle, because matrix structure, opacity, fat content, turbidity, and organic matter may affect dye penetration and photoactivation. PMA preferentially enters cells with compromised membranes and binds to accessible DNA after photoactivation, thereby reducing its subsequent PCR amplification. However, PMA-based methods primarily differentiate cells according to membrane integrity rather than directly measuring metabolic activity. PMA-treated sequencing libraries should therefore be interpreted as profiles enriched in DNA from cells with intact membranes, not as an unequivocal representation of the viable and metabolically active microbiome. The effectiveness of PMA exclusion should be verified using appropriate live-cell, dead-cell, and matrix-specific controls.

### 9.4. Multidimensional Data Integration and Chemometric Analysis

**Figure 1:**
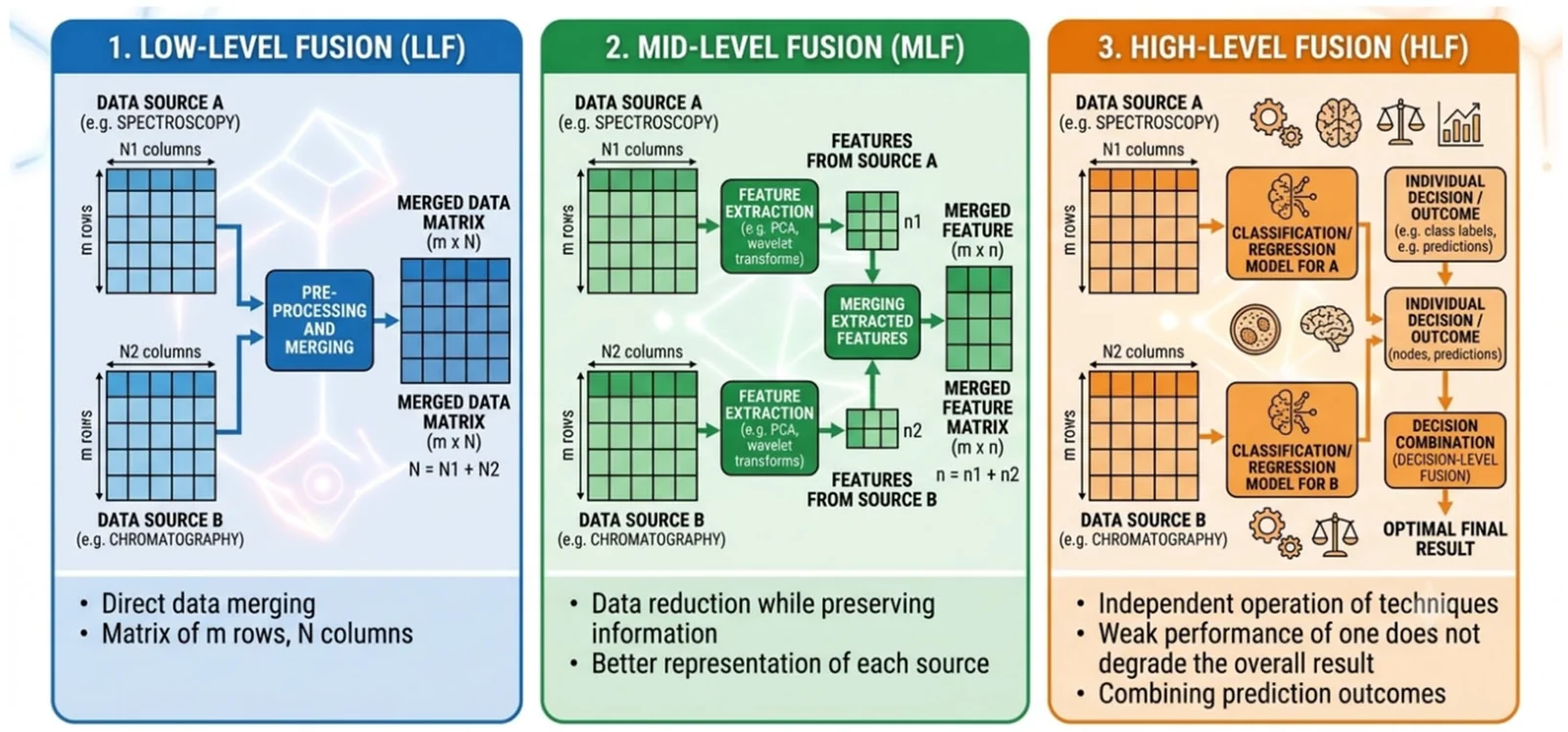
Three levels of data fusion: low-level fusion (LLF), combining preprocessed variables; mid-level fusion (MLF), combining selected or extracted features; and high-level fusion (HLF), combining outputs from independently developed models.

Understanding the interactions between CP and the meat matrix requires the integration of structural, biochemical, and microbiological data, as well as chromatographic, spectroscopic, and sensory measurements. Individual analytical techniques provide only partial insights into these complex processes. Data fusion involves combining information obtained from different analytical sources to provide a more comprehensive characterization of the analyzed matrix. When appropriately implemented, this approach may improve sample discrimination, prediction accuracy, and interpretation by capturing complementary information that is not available from any individual technique [232,233,234,235]. Recent applications in food analysis have shown that multiblock approaches can integrate spectroscopic, metabolomic, physicochemical, and sensory measurements obtained from the same samples [233,235]. However, the reliability of data-fusion models depends on the quality and comparability of the input data. Therefore, preprocessing procedures, including baseline correction, noise reduction, peak alignment, signal transformation, missing-value handling, scaling, and normalization, should be selected according to the characteristics of each analytical platform and described in sufficient detail [236]. Because chromatographic, spectroscopic, microbiological, and sensory datasets may differ substantially in dimensionality, variance, units, and measurement scale, appropriate normalization or block scaling is required to prevent a dataset containing more variables or greater total variance from dominating the fused model [234]. This methodology distinguishes three principal levels of multi-source information fusion: low-level fusion (LLF), mid-level fusion (MLF), and high-level fusion (HLF) (Figure 1) [233,235]. These strategies differ according to whether raw or preprocessed variables, extracted features, or outputs of separately developed models are combined [223,234].

LLF is obtained by combining preprocessed data from individual sources into a single matrix composed of m rows, representing individual samples, and N columns, representing measurement variables from all analytical sources. LLF retains much of the original information but may generate a high-dimensional matrix containing redundant, collinear, or irrelevant variables. It is therefore particularly sensitive to differences in data scale and generally requires consistent preprocessing, block normalization, and careful variable selection [234]. MLF involves extracting relevant features separately from the data generated by each analytical technique and subsequently combining these features into a single matrix. Feature extraction or selection reduces data dimensionality while preserving the most informative variation and allows each analytical source to be represented more appropriately than in LLF [234]. Potential features may include selected chromatographic peaks, spectral bands, principal-component scores, microbial counts or taxa, oxidation markers, and sensory attributes. Multiblock food-analysis studies have demonstrated that metabolomic fingerprints, NIR spectra, quality parameters, and sensory properties can be analyzed jointly to identify relationships among complementary datasets [233]. However, the feature-selection procedure and its selection criteria should be explicitly reported. Variable selection is particularly important for high-dimensional analytical datasets in which only a subset of the measured variables may contain useful information [237]. To avoid information leakage and overly optimistic estimates of model performance, supervised variable selection, normalization, and parameter optimization should be conducted using only the training data within the model-validation procedure [237,238]. HLF, also referred to as decision-level fusion, combines the outputs of classification or regression models developed independently for individual analytical techniques. These outputs may include predicted classes, class probabilities, model scores, or continuous response values. Because each data source is modeled separately, HLF can accommodate datasets with different structures, dimensionalities, and preprocessing requirements [234]. Nevertheless, weak or biased individual models may still affect the fused decision, depending on the weighting, voting, or aggregation strategy. Therefore, the method used to combine individual model outputs should be clearly specified and validated rather than assuming that poor performance from one analytical technique cannot reduce overall model performance [234]. Regardless of the fusion level, model development should include appropriate internal and, where possible, external validation. Training, validation, and test sets should be independent at the level of biological batches, production lots, animals, storage experiments, or measurement sessions rather than being created solely by randomly separating technical replicates. External validation is particularly valuable because reliance exclusively on internal cross-validation may produce overly optimistic estimates of model performance [238]. Cross-validation procedures, performance metrics, class balance, sample size, and the number of retained variables or latent components should therefore be reported transparently [236,238]. Repeated cross-validation, nested cross-validation, permutation testing, or an independent test set may be used to assess model robustness and reduce the risk of overfitting. Preprocessing, variable selection, and hyperparameter optimization should be performed within the cross-validation loop rather than on the complete dataset before its division into training and test sets. This approach prevents information from validation or test samples from inadvertently influencing model development [237,238]. Model performance should also be compared with that of models based on individual analytical techniques to demonstrate whether data fusion provides a genuine analytical advantage. Food-authentication studies integrating data from complementary analytical platforms illustrate how fusion models can be compared with single-platform models and independently tested for classification purposes [233,239]. Careful interpretation is particularly important when the number of variables substantially exceeds the number of independent samples, as commonly occurs when chromatographic and spectroscopic fingerprints are combined. Dimensionality reduction and variable selection should therefore be optimized within the validation framework rather than on the complete dataset [236,237,238]. In addition to predictive accuracy, the stability and biological relevance of selected variables should be examined to determine which chemical, microbiological, structural, or sensory changes contribute to the differentiation of CP-treated meat samples.

Visualizing and interpreting such large and heterogeneous datasets requires the use of dimensionality-reduction, variable-projection, and multivariate modeling methods. However, unsupervised methods, such as principal component analysis, used for data exploration should be clearly distinguished from supervised methods used for classification or quantitative prediction. Visual separation of samples in an exploratory score plot should not be treated as evidence of predictive performance without appropriate model validation [236,240].

Visualizing and interpreting such large and complex datasets requires dimensionality-reduction, variable-projection, and multivariate modeling methods:

PCA (Principal Component Analysis): PCA is an unsupervised exploratory method that reduces data dimensionality and allows visualization of sample grouping according to plasma-processing parameters. It can also identify the variables that contribute most strongly to overall sample variability, such as discharge voltage, exposure time, and working-gas composition, and may help distinguish patterns associated with exposure time from those associated with plasma-generator power [183]. However, PCA does not provide a validated classification model, and visual separation of samples in a score plot should not be interpreted as evidence of statistically significant group differences or predictive performance.

PLS-DA (Partial Least Squares Discriminant Analysis): PLS-DA is a supervised method used to develop classification models and identify variables that contribute to group discrimination. Variable Importance in Projection (VIP) scores can be used to evaluate the contribution of individual variables to the PLS-DA model. The method may also support the prediction of meat-quality characteristics, such as drip loss, based on FTIR spectra and electrophoretic profiles [201]. Variables with VIP values above 1 may contribute substantially to group discrimination, but this threshold alone does not demonstrate that they are specific or unambiguous oxidative markers of CP treatment. Their relevance should be confirmed through validated analytical identification, appropriate univariate statistical testing, and correction for multiple comparisons. Where possible, the selected variables should also be verified in an independent sample set and interpreted in relation to established biochemical or chemical indicators of oxidation. In addition, the stability of VIP scores should be assessed across model resampling or cross-validation procedures because variable importance may depend on sample composition and model specification [193].

Variables with VIP values > 1.0 may contribute substantially to group discrimination in a PLS-DA model but should not automatically be classified as specific or unambiguous oxidative markers induced by CP treatment. Candidate markers require confirmation through validated analytical identification, appropriate univariate testing, and correction for multiple comparisons. The reliability of the PLS-DA model should be documented by reporting R^2^X, R^2^Y, and Q^2^ values, together with the cross-validation strategy and the results of permutation testing. Whenever possible, predictive performance and the stability of the selected variables should also be evaluated using an independent external validation set [201]. These tools may support the identification of an optimized processing window that maximizes microbial inactivation while limiting undesirable physicochemical, nutritional, and sensory changes.

## 10. Future Research Directions

Multi-hurdle strategies represent an important direction for future research and should combine ACP or PAW with natural antioxidants, active coatings, modified-atmosphere packaging, or other preservation technologies. These combinations may improve microbial control while limiting oxidative changes and preserving meat quality. However, their effectiveness should be comprehensively evaluated using microbiological, chemical, sensory, and toxicological endpoints. In parallel, data-fusion approaches integrating HS-SPME-GC-MS, GC-IMS, and electronic-nose measurements with appropriately validated chemometric models, such as PCA and PLS-DA, may support the identification of an optimized processing window. Such an operating window should balance pathogen inactivation with the physicochemical and sensory quality and toxicological safety of meat. However, it should not be regarded as a universally applicable “sweet spot,” because optimal treatment conditions may depend on the meat matrix, plasma source, process parameters, packaging system, and storage conditions. Optimization of IPCP systems combined with MAP should consider gas composition, electrode geometry, absorbed power, treatment time, package atmosphere, reactive-species profile, product geometry, and matrix composition. Laboratory-scale findings cannot be linearly extrapolated to conveyor-based or industrial in-package systems because changes in product geometry and throughput, electrode configuration, treatment uniformity, shadow effects, reactive-species generation, lifetime and transport, package headspace, and interactions with packaging materials may substantially modify both microbial efficacy and physicochemical effects. Consequently, industrial scale-up requires system-specific reactor redesign, product-specific optimization, and pilot-scale validation rather than the proportional enlargement of laboratory devices. Chemical safety assessment should include potentially harmful compounds, such as N-nitrosamines in cured meats, as well as the stability of plasma-activated solutions and powders.

From a sustainability perspective, future research should verify the environmental and economic performance of both ACP and PAW systems in comparison with conventional preservation technologies, including thermal processing and high-pressure processing (HPP). Although ACP and PAW are generally described as non-thermal technologies, their actual advantages should be assessed using standardized indicators such as specific energy consumption (kWh/kg product), microbial log reduction per unit of energy, process throughput, gas consumption, water demand, and operating cost, together with life-cycle assessment. Such comparisons are particularly important for meat applications because process configuration, packaging mode, treatment time, absorbed power, product geometry, PAW generation and storage conditions, and scale of operation may substantially affect resource consumption, economic feasibility, and industrial scalability.

Future research should include interlaboratory validation trials to establish standardized CP protocols. Harmonized reporting of plasma configuration, gas composition, voltage, current, absorbed power, exposure time, source-to-sample distance, treatment and sample temperature, matrix characteristics, and microbiological and quality endpoints is required to improve reproducibility and enable reliable cross-study comparison. Such validation and reporting standards may also support regulatory assessment and industrial scale-up.

Future research should move beyond the immediate quantification of N-nitrosamines and address the broader long-term dietary safety of meat products modified by RONS. Although monitoring N-nitrosamines remains essential, toxicological assessments should also examine plasma-derived lipid- and protein-oxidation products, including their formation, persistence, transformation, and bioaccessibility during storage, cooking, and gastrointestinal digestion. More evidence is required to identify these compounds and determine whether digestive conditions generate additional oxidation or nitrosation products that are absent immediately after plasma treatment.

Future studies should therefore combine targeted and untargeted chemical profiling with in vitro digestion, bioaccessibility assessment, and appropriate toxicological assays. Realistic repeated dietary-exposure models are also required to determine whether plasma-induced products persist, undergo further transformation, accumulate in biologically relevant forms, or produce adverse effects during long-term consumption.

## 11. Material and Methods

A comprehensive review was conducted to collect and critically analyze studies addressing the application of cold plasma and plasma-activated water in meat and meat products. Literature searches were conducted in the Web of Science Core Collection, Scopus, PubMed, and ScienceDirect databases using database-specific combinations of keywords and Boolean operators. The core search strategy combined terms describing plasma-based technologies, meat matrices, and the main outcome domains as follows: (“cold plasma” OR “cold atmospheric plasma” OR “non-thermal plasma” OR “plasma-activated water” OR PAW) AND (meat OR poultry OR chicken OR beef OR pork OR lamb OR sausage OR ham OR “meat products”) AND (microbial OR quality OR oxidation OR protein OR lipid OR volatile OR analytical). The search syntax was adapted to the indexing rules and field codes of each database. The Scopus strategy was constructed using TITLE-ABS-KEY, which searches titles, abstracts, author keywords, and indexed keywords. The Web of Science Core Collection strategy used TS to search topic-related bibliographic fields, including titles, abstracts, and keywords. In PubMed, plasma- and meat-related expressions were searched using the [tiab] tag, with MeSH terms added only when an appropriate controlled-vocabulary term was available. In ScienceDirect, the equivalent Boolean expression was entered in the Title, abstract, or author-specified keywords field. Database-specific phrase-searching, truncation, field-tag, and filtering conventions were applied.

The literature search was conducted between January and 20 August 2026. Publications from 2016 to 2026 were considered, with explicitly justified exceptions for earlier foundational studies. Eligible publications were peer-reviewed original research articles in English that evaluated direct ACP treatment, in-package plasma treatment, or PAW application in raw, processed, or ready-to-eat meat products and reported microbiological, physicochemical, molecular, sensory, or storage-related outcomes. PAW studies were included when plasma-generated reactive species were transferred to an aqueous medium subsequently applied to meat products. Studies were excluded when they focused exclusively on non-meat matrices, medical plasma applications, animal feed, packaging materials without meat application, or theoretical mechanisms without relevant experimental data. Conference abstracts, editorials, non-peer-reviewed publications, and articles without accessible full text were also excluded. Study selection was conducted in two stages: title and abstract screening followed by full-text eligibility assessment. Database searches yielded 1693 records. After removing 141 duplicates, 1552 unique records remained. Prior to the formal assessment of titles and abstracts, 924 records were excluded during the initial eligibility screening because they fell outside the established timeframe, were of an ineligible document type, or were clearly outside the scope of the review. Consequently, 628 records were assessed based on their titles and abstracts. Of these, 290 were excluded for failing to meet the established eligibility criteria, and the full texts of 338 publications were subsequently evaluated. Following the full-text analysis, 95 publications were excluded, and 240 studies were included in the final qualitative synthesis (Supplementary Materials: Figure S1). Owing to the methodological heterogeneity of the included studies, no overall quality score was assigned. Instead, methodological quality was considered narratively using predefined criteria, including control-group adequacy, replication, completeness of plasma-source and treatment-parameter reporting, meat-matrix characterization, storage conditions, analytical methods, statistical analysis, and completeness of outcome reporting. The absence of a standardized formal risk-of-bias assessment should be considered a limitation of this review. Zotero 9.0.5 was used for reference management and initial duplicate identification. Potential duplicates were manually verified by comparing titles, authors, publication years, journal details, and DOI numbers. Records with incomplete or inconsistent metadata were checked against the original publications, and only confirmed duplicates were merged. The remaining records were additionally screened by title and DOI to identify duplicates not detected automatically.

## 12. Conclusions

A comprehensive narrative review was conducted to examine the applications of cold plasma and plasma-activated water (PAW) in meat and meat products. Relevant publications were identified through searches of the Web of Science Core Collection, Scopus, PubMed, and ScienceDirect databases. The core search strategy combined terms describing plasma-based technologies, meat matrices, and the principal outcome domains as follows: (“cold plasma” OR “cold atmospheric plasma” OR “non-thermal plasma” OR “plasma-activated water” OR PAW) AND (meat OR poultry OR chicken OR beef OR pork OR lamb OR sausage OR ham OR “meat products”) AND (microbial OR microbiological OR quality OR physicochemical OR oxidation OR oxidative OR protein OR lipid OR volatile OR sensory OR color OR color OR texture OR storage OR “shelf life” OR safety OR analytical).

The search syntax was adapted to the indexing rules and field codes of each database. In Scopus, the TITLE-ABS-KEY field was used to search titles, abstracts, author keywords, and indexed keywords. In the Web of Science Core Collection, the TS field was used to search titles, abstracts, author keywords, and Keywords Plus. In PubMed, plasma- and meat-related expressions were searched primarily using the [tiab] field tag, with controlled-vocabulary terms added where appropriate. In ScienceDirect, the corresponding Boolean expression was entered in the title, abstract, or author-specified keywords field. Database-specific conventions concerning phrase searching, truncation, field tags, and filters were applied. The complete database-specific search strategies are provided in Table S1. Figure S1 presents the flowchart of the literature search and publication selection process for the narrative review.

The literature searches were conducted from January 2026 to 20 August 2026, which was the final search date, and primarily covered English-language publications published between 2016 and 2026. Publications predating 2016 were considered when they provided essential methodological, mechanistic, or historical context. Reference lists of relevant original and review articles were additionally examined to identify potentially relevant publications not retrieved through the database searches. Peer-reviewed original research articles were considered for the primary narrative synthesis when they evaluated direct atmospheric cold plasma treatment, in-package plasma treatment, or PAW application in raw, processed, or ready-to-eat meat products and reported microbiological, physicochemical, molecular, oxidative, sensory, or storage-related outcomes. Publications focused exclusively on non-meat matrices, medical plasma applications, animal feed, packaging materials without direct application to meat, or theoretical mechanisms without relevant experimental data were excluded from the primary evidence synthesis. Review articles, methodological references, and foundational publications were used for contextual support and to identify additional original studies.

The identified publications were initially assessed for thematic relevance based on their titles and abstracts, followed by full-text evaluation of potentially relevant articles. Data were extracted from the full texts, tables, figures, and Supplementary Materials using a consistent thematic framework. The extracted information included the meat matrix, plasma source, working gas, voltage or power, treatment duration and application mode, storage conditions, analytical methods, and reported microbiological, physicochemical, oxidative, molecular, and sensory effects.

Owing to substantial heterogeneity in plasma-generation systems, treatment conditions, meat matrices, storage protocols, analytical methods, and outcome reporting, the evidence was synthesized narratively rather than quantitatively pooled. Greater interpretive emphasis was placed on studies employing appropriate controls and experimental replication and providing clearly reported plasma parameters, adequately characterized meat matrices, and quantitative outcome data. Of the 240 references cited in the manuscript, 51 original research articles directly contributed data to the narrative synthesis. The remaining references comprised review articles, methodological sources, and foundational publications used for mechanistic, historical, or contextual support.

## Figures and Tables

**Table 1:** Cold plasma sources, methods of their electrical excitation and modes of application in the processing of meat matrices.

Plasma Source Type	Typical Electrical Excitation	Application Mode
Dielectric Barrier Discharge, DBD	Alternating or pulsed voltage, usually high voltage with a frequency of Hz to kHz	Direct, indirect or inside the package
Atmospheric Plasma Jet, APPJ	Alternating, pulsed or radio frequency voltage	Direct or indirect; nozzle directed at the product surface
Crown discharge	High DC, AC or pulse voltage between the point electrode and the counter electrode	Direct or indirect
Gliding arc	High alternating or pulsed voltage; discharge channel moved by gas flow	Most often, an intermediate product; the product is subjected to post-plasma gas.
Low-pressure glow discharge	DC, AC or radio frequency field	Direct, in a closed vacuum chamber
Microwave-excited plasma	A microwave field, often at a frequency of around 2.45 GHz	Direct or indirect, at reduced or atmospheric pressure

**Table 2:** (**A**) Principal cold-plasma source types used or investigated in food-processing applications. Sources: [39,40,43,55,56,57,63,64,65,75]. (**B**) Application modes of cold plasma and plasma-activated media in meat systems.

(**A**)						
Plasma-source type	Basic discharge characteristics	Possible implementation layouts	Common feed gases	Relevance to meat processing	Main limitations	
Dielectric barrier discharge, DBD	Discharge generated between electrodes separated by at least one dielectric barrier; it may operate in filamentary, diffuse, or mixed modes	Direct treatment, remote treatment, surface DBD, volume DBD, in-package DBD, conveyor-compatible systems	Air, N_2_, O_2_, Ar, He, and gas mixtures	Surface decontamination, in-package treatment, packaging modification, direct treatment of meat and meat products	Treatment uniformity depends on electrode geometry, product shape, discharge gap, packaging properties, humidity, and gas composition	
Corona and pulsed-corona discharge	Localized discharge generated near a high-curvature electrode under a strong, non-uniform electric field	Direct, non-contact, remote, or flow-through treatment	Air and other gases or gas mixtures	Surface decontamination and treatment of packaging or process gases	Localized treatment, possible non-uniformity, and sensitivity to electrode–sample distance	
Atmospheric-pressure plasma jet (APPJ)	Plasma generated in a flowing feed gas and transported beyond the electrode region as a directed effluent or plume	Direct or remote localized treatment; single or multiple jets	He, Ar, air, N_2_, and mixtures containing O_2_ or water vapour	Localized surface treatment and experimental processing of irregular surfaces	Limited treatment area, possible shadowing, gas consumption, and changes in effluent chemistry caused by ambient-air entrainment	
Atmospheric-pressure glow discharge	Diffuse non-equilibrium discharge operated under conditions that limit transition to filamentary or arc regimes	Direct or non-contact treatment	He, Ar, N_2_, air, or gas mixtures, depending on source design	Relatively uniform treatment where a diffuse discharge can be maintained	Source-dependent stability; transition to filamentary or thermal regimes may occur when operating conditions change	
Radio-frequency or microwave plasma source	Plasma sustained by radio-frequency or microwave electromagnetic excitation	Direct, remote, atmospheric-pressure, or low-pressure configurations	Ar, He, N_2_, O_2_, air, and gas mixtures	Surface modification, decontamination, and plasma-liquid activation	Not all RF or microwave plasmas are cold; the non-thermal regime and gas or product temperature must be demonstrated	
Gliding arc	Discharge formed between diverging electrodes and displaced by gas flow	Direct or remote flow-through treatment	Air, N_2_, CO_2_, and gas mixtures	Potential generation of high concentrations of reactive species	May operate in transitional or thermal regimes; classification as CP requires experimental verification of the operating regime	
(**B**)						
Application mode	Location of plasma generation and interaction	Compatible plasma sources	Typical applications in meat processing	Main advantages	Principal limitations or quality risks	Sample sources
Direct plasma treatment	Plasma is generated in direct contact with, or immediately adjacent to, the product surface	DBD, plasma jet, corona, glow discharge	Rapid surface decontamination and modification of surface properties	Direct delivery of reactive species, charged particles, photons, and electric fields	Local oxidation, myoglobin modification, surface drying or etching, and treatment non-uniformity	[21,55,58,76,77,78,79,80,81]
Remote or indirect gas-phase treatment	Plasma is generated away from the product, and mainly the plasma effluent or longer-lived species reach the surface	Plasma jet, remote DBD, corona, RF or microwave source	Decontamination of heat-sensitive or geometrically complex products	Reduced exposure to the discharge zone and potentially lower contribution of charged particles or electric fields	Loss of short-lived species during transport; strong dependence on distance, gas flow, and ambient-air mixing	[58,66,70,71,72,73,74]
In-package cold plasma, IPCP	Plasma is generated inside a sealed or semi-sealed package containing the product	Most commonly DBD; other source designs may also be possible	Decontamination after packaging and extension of refrigerated shelf life	Reduced risk of post-treatment recontamination; use of package headspace as the working atmosphere	Strong dependence on packaging material, headspace composition and volume, product geometry, voltage or power, and post-treatment storage time; oxidation may occur	[56,60,82,83,84,85,86,87,88]
Plasma-activated water or plasma-treated water (PAW/PTW)	Plasma is first applied to water; the resulting liquid is subsequently used by spraying, washing, dipping, thawing, or as a formulation ingredient	DBD, plasma jet, corona, or other gas–liquid plasma configurations	Decontamination, thawing, curing, and transfer of long-lived reactive species	Separation of plasma generation from product treatment; adaptable to existing liquid-processing operations	Composition changes during storage; requirements for pH, NO_2_^−^, NO_3_^−^, H_2_O_2_, ORP, and chemical-safety control	[47,50,89,90,91,92,93,94]
Liquid plasma immersion	The discharge interacts with a liquid containing or contacting the product	Submerged, liquid-surface, or gas–liquid discharge systems	Washing and microbial decontamination	Combined liquid contact and plasma-derived antimicrobial effect	Process classification and liquid chemistry depend strongly on reactor design; possible oxidative changes	[95]
Plasma-assisted curing	Reactive nitrogen species are generated directly in the product, brine, water, or another curing carrier	Direct DBD, PAW/PTW, plasma-treated brine, or plasma-activated ingredients	In situ generation or delivery of nitrite-related curing functionality	Potential reduction of directly added conventional nitrite and development of cured-meat color	Variability of nitrite and nitrate concentrations, matrix-dependent curing response, and requirement for N-nitrosamine validation	[63,89,92,96,97,98,99]
Plasma-activated powders or solutions	A solid or liquid carrier is plasma-treated before incorporation into the meat formulation	DBD, plasma jet, or another suitable source	Alternative curing ingredients and delivery of plasma-generated reactive compounds	Plasma treatment can be performed separately from meat processing; easier formulation control	Batch variability, stability of reactive compounds, sensory effects, labelling, and toxicological validation	[96,98,99,100,101]
Plasma combined with packaging	CP is combined with modified-atmosphere packaging, edible coatings, essential oils, UV, or other preservation strategies	DBD, in-package DBD, plasma jet, or remote plasma systems	Enhanced microbial control while reducing the required intensity of individual treatments	Potential synergistic effect and improved balance between microbial safety and product quality	Interactions between treatments, migration from packaging, oxidation, sensory changes, and increased process complexity	[102,103,104,105,106,107]

HVACP/HV-CAP is not treated here as a separate plasma-source category. It describes an atmospheric cold-plasma operating configuration using high voltage and may be implemented through different source designs, most commonly DBD, as well as through direct, remote, or in-package treatment layouts. Similarly, “in-package plasma” describes the location and mode of plasma treatment rather than the underlying discharge type.

**Table 3:** CP process parameters and their directional influence on microbiological effectiveness and meat quality. Note: The effects of increasing exposure time, voltage, or power should not be interpreted as strictly linear. RONS generation and microbial inactivation may increase only within an optimal operating window and may plateau or change due to gas heating, ozone decomposition, and plasma-chemistry mode transitions. In ACP and PAW treatments, uniformity and effectiveness also depend on product geometry, surface irregularities, plasma-source configuration, and exposure conditions.

Process Parameter	Typically Observed Effect on Microorganism Reduction	Quality Risk	Design/Interpretation Notes	Sources
Exposure time (↑)	May increase inactivation within an optimal treatment window; plateau or diminishing returns may occur	Longer exposure may increase TBARS, VOC changes, and color alterations	Longer treatment does not necessarily imply proportional RONS increase. Extended operation may change reactor temperature and gas-phase chemistry; therefore, exposure time should be optimized rather than maximized	[80,82,83,84,119]
Voltage/Power (↑)	May increase inactivation when operating within the non-thermal and chemically favourable regime	Higher power/voltage may increase oxidation and color changes	The effects of increasing voltage or power are not necessarily linear. Depending on the plasma source and operating conditions, higher energy input may alter gas temperature and reactive-species composition rather than proportionally increasing ROS and RNS generation	[55,78,85,86,119]
Gas composition O_2_/air	Often effective due to ROS/RNS formation, but the dominant species depend on operating conditions	Higher O_2_/air reactivity may increase TBARS, aldehydes/ketones, and met-Mb formation	Oxygen-containing atmospheres can promote ozone formation, but ozone concentration is temperature- and mode-dependent; heating can accelerate ozone loss and shift chemistry toward nitrogen oxides	[46,56,76,80,119,120]
Product temperature/reactor heat load	Can indirectly affect inactivation by changing the concentration and lifetime of reactive species	Increased temperature may intensify quality changes and alter reactive-species profiles	CP is non-thermal with respect to the product, but reactor/gas heating may occur. Temperature should be monitored because it can affect ozone decomposition and plasma-chemistry mode transitions	[13,36,119]
Product geometry, surface irregularities, and plasma-source configuration	May cause spatially non-uniform microbial inactivation because reactive species and plasma-generated effects may not reach all areas equally	Overexposure of directly treated regions may increase local oxidation or color changes, while recessed or shadowed areas may remain insufficiently treated	Treatment uniformity and effectiveness should be assessed for the actual product shape and surface. Source configuration, treatment distance, product orientation, and exposure conditions should be optimized and reported; ACP and PAW should not be assumed to provide homogeneous decontamination	[60,108,121,122,123]

↑ increase.

**Table 4:** Applications of ACP and PAW in Different Meat Products: Reported Effects and Critical Limitations.

Matrix/Product	Technological Goal	Documented Effects (General)	The Most Common Quality Risks	Sample Studies
Poultry (breast/fillets, RTE)	reduction in *Salmonella*, *E. coli*, extension of shelf life	Uncoated chicken breast cubes: 0.8 ± 0.3 log CFU/sample WPI-coated cubes: 1.5 ± 0.3 log CFU/sample; reduction in psychrophiles, *Campylobacter*, and *Salmonella* by up to 90% on the surface of raw meat	color changes (a*), gas and voltage dependent; potential VOC	[60,78,82,83,84,85,88]
Beef (cutlets, RTE, processed products)	safety + oxidation and color control	pathogen reduction; possible changes in myoglobin and TBARS	browning (met-Mb), increase in TBARS and aldehydes	[76,77,120,127]
Pork (tenderloin, RTE ham, MAP)	decontamination + durability in MAP	limitation of micro-flora in MAP; dependence on parameters	TBARS under intense conditions; VOC	[56,122,123,128]
Dried products/jerky	control of mold and microflora, improvement of safety in packaging	efficacy in packaging; dose-dependent stability	possible increase in undesirable odors with strong processing	[92,126,129]
Cold cuts/cured products (ham/sausage)	nitrite reduction, clean label curing, color	Taste and overall acceptability scores for the ham were 5.06 and 5.26, respectively, for APP, and 3.95 and 4.31 for the sodium nitrite-cured product.	risk of N-nitrosamines (RNS dependent); need for process control	[63,89,92,96,98,99,100]

APP—atmospheric pressure plasma; MAP—modified-atmosphere packaging; RTE—ready-to-eat products; TBARS—secondary lipid oxidation product index; WPI—whey protein isolate.

**Table 5:** Matrix- and Treatment-Specific Chemical and Quality Risks Associated with ACP and PAW Treatment of Meat Products.

Reported Risk	Matrix/Product	Plasma Conditions	Analytical Marker	Magnitude of Change	Proposed Mitigation Strategy	Sources
Lipid oxidation	High-fat meat products; minced beef, lamb, pork, and chicken breast; pork loin; various foods; various meat products; beef meatballs with beef or sheep-tail fat	In-package DBD, ambient air, 80 kV, 10 min; in-package DBD, MAP (20–60% O_2_, 20% CO_2_, balance N_2_), 85 kV, 60 s; various ACP systems and working gases;	TBARS, lipid hydroperoxides, hexanal and other oxidation-derived VOCs	TBARS increased significantly in plasma-treated lamb and pork after 24 h and 7 days and in beef after 24 h; In pork loin packaged under 60% O_2_ MAP, TBARS increased significantly on days 8 and 12 (*p* < 0.05), but exact values and percentage changes were not reported.	Reduce treatment intensity; minimize oxygen exposure; use Ar or N_2_ where appropriate; combine with validated antioxidants or oxygen-barrier packaging	[46,56,80,120]
Red-meat discoloration	Beef patties; various fresh and processed meat products; fresh red meat	Direct DBD-CP, ambient air, 50–70 kV at 50 Hz or 60 kV at 50–110 Hz, 120 s; storage at 4 °C for 8 days; other meat sources reviewed various ACP conditions	L*, a*, b*, ΔE*; oxy-Mb, deoxy-Mb and met-Mb	Significant decrease in redness (a*) and increase in metmyoglobin in plasma-treated beef patties (*p* < 0.05); numerical magnitude not reported.	Reduce exposure time or energy; control oxygen and storage conditions; optimize the gas composition	[76,120,166,167,168]
Volatile-profile alteration	Various meat products; meatballs; myofibrillar proteins from dry-cured bacon; roasted lamb and RTE meat products	Various ACP conditions; Ar-based MAP, 3–9 min, refrigerated storage for 14 days; DBD-CP, 50–70 kV, 150 s, storage not applicable; gas not reported.	GC-MS, GC-IMS, electronic nose, sensory analysis	Altered volatile profile and increased binding of selected aldehydes at 50–70 kV; overall quantitative magnitude not reported	Optimize treatment dose and headspace composition; use validated antioxidants; verify sensory acceptance	[120,122,154,177]
N-nitrosamine formation	Cured ground ham; various foods and meat products	RNS-generating plasma, PAW/PTW or plasma-treated brine; ACP-treated cauliflower powder; 60 or 100 mg/kg initial nitrite; complete ACP and storage parameters not reported	Individual volatile and non-volatile N-nitrosamines; residual NO_2_^−^ and NO_3_^−^	Significant reduction in NPYR at 100 mg/kg initial nitrite (*p* < 0.05); numerical magnitude not reported	Control plasma-generated RNS and residual nitrite; optimize curing and cooking; validate N-nitrosamines in each product	[2,6,100]
Loss of protein functionality	Protein ingredients; intact beef; comminuted beef products.	Various CAP conditions; direct DBD-CP, air, 80 kV, 15 min; in-package CP pretreatment or post-roasting CP treatment, parameters study-dependent.	Carbonyls, sulfhydryl groups, solubility, aggregation, hydrophobicity, gel strength, water-holding capacity	Treatment-dependent protein unfolding, oxidation, and aggregation may impair techno-functional properties; no single quantitative magnitude was reported	Establish a matrix-specific treatment optimum; reduce exposure when aggregation or solubility loss occurs	[143,145,172,173]

**Table 6:** Matrix-Specific Oxidative Risks, Their Magnitude, and Mitigation Strategies in Cold-Plasma-Treated High-Fat Meat Products.

Matrix/Product	Plasma Conditions	Reported Risk	Analytical Marker	Magnitude and Direction of Change	Proposed Mitigation Strategy	Sources
Chicken breast	Atmospheric CP, 100 kV, 1–5 min	Potential lipid oxidation	TBARS or marker used in the original study	No statistically significant change during short exposure	Maintain short exposure; verify oxidation during storage before increasing treatment time	[83]
Marinated poultry meat with essential oil	Combined CP and marinade treatment; complete parameters should be added; Custom-made CP; 32 kHz, 10 min; Ar (99.997%) at 6 L/min; voltage and power not reported	Storage-related lipid oxidation despite the combined treatment	TBARS	Combined ACP + ZEO + AEO + CEO reduced *S. aureus* and *E. coli* counts by 4.1 and 4.7 log CFU/g, respectively, after 14 days, while increasing DPPH radical-scavenging activity from 5.5 ± 0.8% to 36.0 ± 12.3%	Optimize CP intensity and antioxidant concentration; monitor oxidation throughout storage	[178]
Beef bastirma	Atmospheric CP; Ar/O_2_ 75:25; 3–5 min	Increased lipid peroxidation	TBARS	Increase from 0.72 to 0.99 mg MDA/kg, approximately 37.5%	Reduce the oxygen fraction; evaluate Ar or N_2_-rich mixtures; shorten treatment while maintaining microbial efficacy	[133]
Pork ham	Atmospheric CP; 300 W, 3500 Hz, 30–180 s	Accelerated oxidative rancidity	TBARS	Increase from 0.92 to 1.53 mg MDA/kg, approximately 66.3%	Reduce treatment duration or energy; use product-compatible antioxidants or oxygen-control packaging	[123]
Pork meatballs	In-package DBD-ACP in Ar	Formation or release of oxidation-related VOCs	Nonanal, (E)-2-octenal, benzaldehyde, 2-hexanol; GC-based analysis	Microbial counts decreased by 1.02–1.19 log CFU/g and shelf life increased by 14 days; lipid oxidation rose slightly from 0.83 to 0.92–0.93 mg/kg, while 9-min treatment significantly altered the odor profile	Limit CP exposure to 6 min; monitor headspace volatiles during storage and validate sensory relevance	[122]
Fresh beef patties	Direct DBD-CP in ambient air; 50, 60, or 70 kV at 50 Hz, or 60 kV at 50, 70, 90, or 110 Hz; 120 s; storage at 4 °C for 8 days	Voltage-dependent lipid and myoglobin oxidation, loss of redness, and reduced metmyoglobin-reducing activity	TBARS (mg MDA/kg), CIELAB a*, OMb%, MMb%, and MRA	After 8 days, increasing voltage from 50 to 70 kV raised TBARS from 1.91 to 3.97 mg MDA/kg, reduced a* by 4.86–6.80 units, increased MMb by 20.8–28.5%, and decreased MRA by 58.7–79.4%; frequency had no significant effect on TBARS or MMb%	Use the lowest effective voltage, preferably 50 kV under the tested conditions, because TBARS remained below the 2.5 mg MDA/kg rancidity threshold after 8 days; optimize voltage rather than frequency and monitor TBARS, a*, MMb%, and MRA during storage	[76]

Essential oils (EOs): saffron essential oil (CEO; *Crocus sativus* L.), garlic essential oil (AEO; *Allium sativum* L.), and *Zataria multiflora* essential oil (ZEO; *Zataria multiflora* Boiss.).

**Table 7:** Proposed and Experimentally Evaluated Strategies for Mitigating Cold-Plasma-Induced Risks in Meat Products.

Target Risk	Matrix/Product	Plasma or PAW Conditions	Marker Triggering Mitigation	Reported Magnitude	Mitigation Strategy	Evidence Status	Sources
Lipid oxidation and VOC formation	High-fat meat products; Various meat products; meatballs; various foods	Direct or in-package CP; Various ACP/DBD conditions; Ar-based MAP, 3–9 min; no PAW treatment	TBARS; hexanal; oxidation-derived VOCs	Microbial counts decreased by 1.02–1.19 log CFU/g and shelf life increased by 14 days; lipid oxidation rose from 0.83 to 0.92–0.93 mg/kg, while VOC changes became significant after 9 min	Replace or reduce O_2_ with Ar/N_2_; shorten exposure	Experimentally evaluated in specified products or proposed, depending on source	[79,120,122]
Recontamination and oxidation during storage	Fresh pork loin; boiled chicken breast; raw chicken breast; RTE chicken breast cubes	In-package CP combined with MAP or vacuum	Microbial counts; TBARS; color; VOCs	Microbial reduction up to 1.7 log; oxidation increased in pork but remained unchanged in RTE chicken	Optimize headspace and barrier properties; use in-package treatment	Product-specific experimental evidence	[56,60,82,88]
Excessive direct-plasma effects	Meat surfaces or thawing/curing systems	PAW/PTW applied by spray, immersion, thawing or curing	Microbial reduction; color; oxidation; texture	Log reduction: 0.71–1.76 log, depending on product; not quantified for loin ham and duck fillets. Quality changes: Generally unchanged or reduced oxidation; product-dependent changes in redness, flavor, and moisture retention.	Use indirect treatment; optimize PAW composition and contact time	Requires product-specific validation	[89,90,91,92,93,94]
Lipid oxidation	High-fat meat products	CP combined with antioxidants, essential oils or edible films	TBARS, peroxide value, volatile aldehydes	After 5 days, TBARS reached 2.90 mg MDA/kg with CP alone versus 2.06 mg MDA/kg with CP + 1% rosemary extract, a reduction of 0.84 mg MDA/kg (29%)	Optimize antioxidant concentration and verify sensory effects	Experimentally evaluated only for specified formulations	[105,177,183,184]
Red-meat discoloration	Beef or other red-meat products	Oxygen-containing plasma; various CP conditions; direct DBD-CP in air, 50–70 kV/50 Hz or 60 kV/50–110 Hz, 120 s, storage at 4 °C for 8 days	L*, a*, b*, ΔE*; oxy-Mb, deoxy-Mb, met-Mb	a* decreased by 4.86–6.80 units and MMb increased by 20.8–28.5%	Reduce oxygen exposure, voltage/power or treatment time	Proposed or experimentally evaluated depending on cited study	[76,166,167,168,179]
Excessive nitrosation or N-nitrosamine formation	Cured meat products	Direct APP curing; PTW curing; air/N_2_ plasma-activated brine, 20 kHz, ~18 h at 4 °C; plasma-treated phosphate solution, 40 W, 30 L/min, 45 min, stored at −20 °C	Residual NO_2_^−^/NO_3_^−^; individual N-nitrosamines	Nitrite increased from 0.64 to 60.50 mg/kg during direct plasma curing; PTW/PAB maintained or reduced residual nitrite, but N-nitrosamines were not measured.	Standardize RNS delivery; control residual nitrite; validate after storage and cooking	Requires product-specific chemical and toxicological validation	[63,89,92,96]
Insufficient microbial reduction at acceptable CP dose	Meat products with coatings, oils or combined barriers	In-package DBD-CP with malic-acid/WPI film; encapsulated DBD plasma + clove oil; DBD-CP on pork loin, up to 70 kV	Log CFU/g or log CFU/cm^2^; TBARS; sensory scores	Microbial reduction: Enhanced *Salmonella* inactivation; >7.5-log reduction with clove oil + plasma in vitro; Clove oil remained stable; pork DBD-CP reduced redness and pH, increased protein oxidation, and improved emulsification and tenderness	Use hurdle technology rather than increasing CP dose	Experimentally evaluated for specific combinations	[102,106,161]

**Table 8:** Comparison of Analytical Platforms for Evaluating Plasma-Induced Changes in Meat.

Parameter/Feature	HS-SPME-GC-MS	GC-IMS	Electronic Nose (MOS)	Sources
Principle of separation	Partition/adsorption on a capillary column	GC separation followed by ion mobility drift separation (CCS/K_0_)	No physical separation (sensor array response)	[183,200,201]
Identification of compounds	Absolute (EI mass spectra + RI indices)	Good (RI bases and K_0_ drift coefficients)	None (classification of odor patterns)	[201,202]
Limit of detection (LOD)	Medium/High (ppb- ppm)	Ultra-high (ppt-ppb)	Low/Medium (ppm)	[183]
Sample analysis time	Long (30–60 min)	Short (10–15 min)	Very short (1–3 min)	[183,200]
Influence of moisture from the sample	Medium (requires optimization of SPME conditions)	Minimal (structurally tolerant)	Very high (water vapor falsifies the reading of MOS sensors)	[200,203]
Key application in CP	Identification of unknown metabolites and markers	Oxidation kinetics monitoring, at-line studies	Quick quality screening, online control	[86,183,188]
Implementation cost	Very high	Medium/High	Low/Medium	[183,200]

## Data Availability

No new data were created or analyzed in this study. Data sharing is not applicable to this article.

## Supplementary Materials

The following supporting information can be downloaded at: https://www.mdpi.com/article/10.3390/molecules31203697/s1, Figure S1. Flow diagram of the literature search and publication selection process for the narrative review; Table S1. Database-Specific Search Strategies.

## Abbreviations

The following abbreviations are used in this manuscript:

AC	alternating current
ACP	atmospheric cold plasma
AEO	garlic essential oil (*Allium sativum* L. essential oil)
APP	atmospheric-pressure plasma
APPJ	atmospheric-pressure plasma jet
Ar	argon
CAP	cold atmospheric plasma
CCS	collision cross section
CD	circular dichroism
CEO	saffron essential oil (*Crocus sativus* L. essential oil)
CFU	colony-forming units
CO_2_	carbon dioxide
CP	cold plasma
Cys	cysteine
DBD	dielectric barrier discharge
DBD-CP	dielectric barrier discharge cold plasma
DC	direct current
deoxy-Mb	deoxymyoglobin
DNA	deoxyribonucleic acid
DNPH	2,4-dinitrophenylhydrazine
DTT	dithiothreitol
EI	electron ionization
EMA	ethidium monoazide
EOs	essential oils
e-nose	electronic nose
FTIR	Fourier-transform infrared spectroscopy
GC	gas chromatography
GC-IMS	gas chromatography–ion mobility spectrometry
GC-MS	gas chromatography–mass spectrometry
H_2_O_2_	hydrogen peroxide
He	helium
HLF	high-level data fusion
HNO_2_	nitrous acid
HNO_3_	nitric acid
HONO	nitrous acid, structural notation
HPP	high-pressure processing
HS-SPME-GC-MS	headspace solid-phase microextraction coupled with gas chromatography–mass spectrometry
HVACP/HV-CAP	high-voltage atmospheric cold plasma
IMS	ion mobility spectrometry
IPCP	in-package cold plasma
IoT	Internet of Things
K_0_	reduced ion mobility
LC-MS/MS	liquid chromatography–tandem mass spectrometry
LLF	low-level data fusion
LOD	limit of detection
Lys	lysine
MAP	modified-atmosphere packaging
MDA	malondialdehyde
Met	methionine
met-Mb	metmyoglobin
MLF	mid-level data fusion
MOS	metal-oxide semiconductor
MUFA	monounsaturated fatty acids
N_2_	molecular nitrogen
N_2_O_4_	dinitrogen tetroxide
N_2_O_5_	dinitrogen pentoxide
NaCl	sodium chloride
NIR	near-infrared spectroscopy
NO	nitric oxide
NO_2_	nitrogen dioxide
NO_2_^−^	nitrite ion
NO_3_^−^	nitrate ion
NO_x_	nitrogen oxides
O_2_	molecular oxygen
O_3_	ozone
•OH	hydroxyl radical
ONOO^−^	peroxynitrite
ORP	oxidation–reduction potential
oxy-Mb	oxymyoglobin
PAH	polycyclic aromatic hydrocarbon
PAH8	sum of eight priority polycyclic aromatic hydrocarbons
PAAA	plasma-activated acetic acid
PAS	plasma-activated solution
PAW	plasma-activated water
PCA	principal component analysis
PCoA	principal coordinates analysis
PCR	polymerase chain reaction
PDMS	polydimethylsiloxane
PLS-DA	partial least squares discriminant analysis
PMA	propidium monoazide
PTW	plasma-treated water
PUFA	polyunsaturated fatty acids
PV	peroxide value
Q^2^	cross-validated predictive ability
RF	radio frequency
RH	relative humidity
RI	retention index
RNA	ribonucleic acid
RNS	reactive nitrogen species
RONS	reactive oxygen and nitrogen species
ROS	reactive oxygen species
RTE	ready-to-eat
R^2^X	proportion of variation explained in the predictor matrix
R^2^Y	proportion of variation explained in the response matrix
SDS-PAGE	sodium dodecyl sulfate–polyacrylamide gel electrophoresis
SPME	solid-phase microextraction
TBARS	thiobarbituric acid-reactive substances
Trp	tryptophan
t-SNE	t-distributed stochastic neighbor embedding
UV	ultraviolet
UVA	ultraviolet A
UVB	ultraviolet B
UVC	ultraviolet C
VBNC	viable but non-culturable
VIP	variable importance in projection
VOCs	volatile organic compounds
WHC	water-holding capacity
WPI	whey protein isolate
ZEO	Zataria multiflora essential oil (Zataria multiflora Boiss. essential oil)
