# Supplementary material for: Cold Atmospheric Plasma and Plasma-Activated Water in Meat Processing: Microbial Safety, Molecular Transformations, Quality Changes, and Analytical Assessment
Source: Molecules. 2026 Oct 15;31(20):3697. doi: 10.3390/molecules31203697 (PMC13707862; doi:10.3390/molecules31203697)
Supplement: Supplementary file 1 [file molecules-31-03697-s001.zip › molecules-4585610-supplementary/Supplementary Materials. Figure S1. Flow diagram(1).pdf]

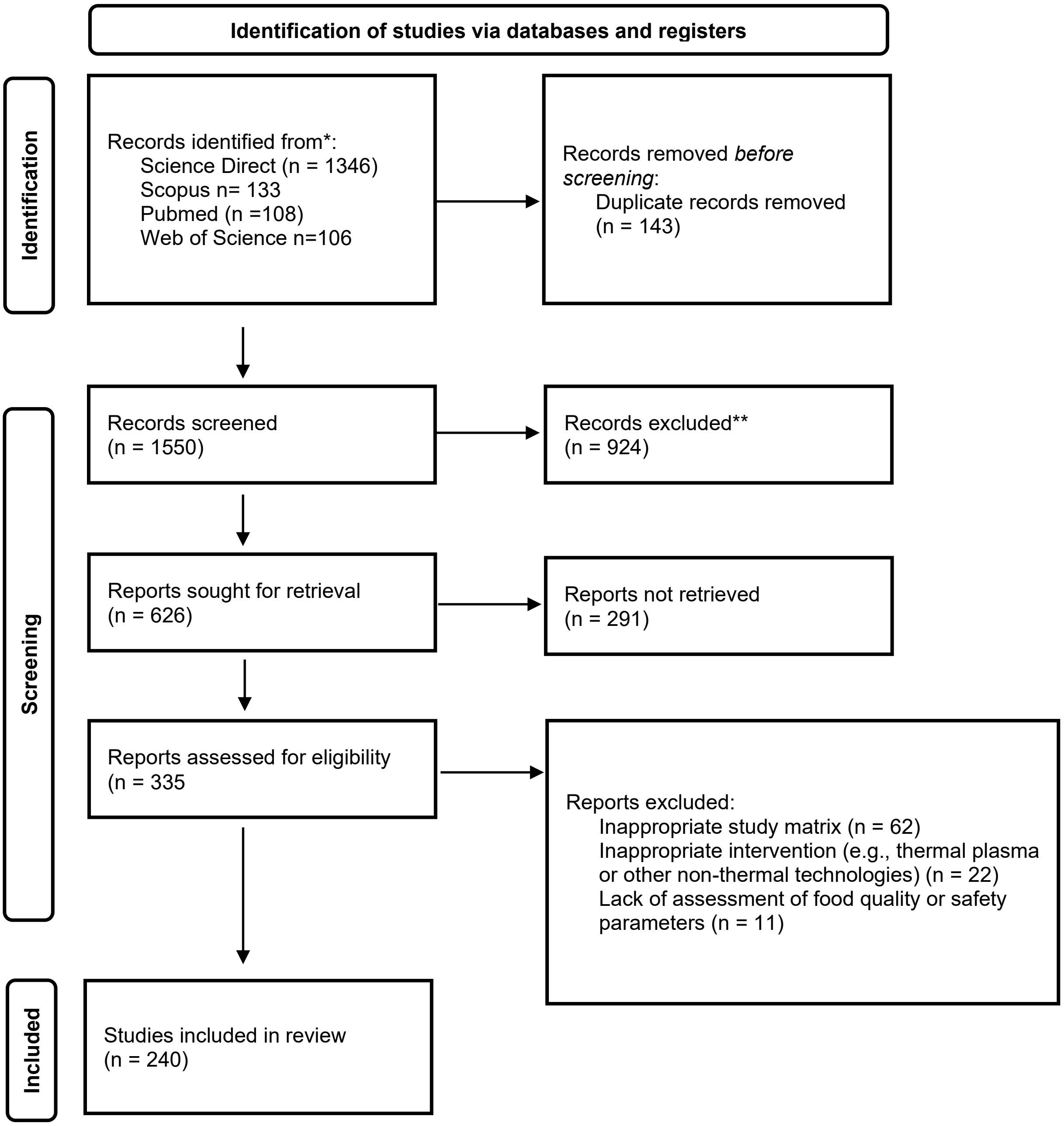

Figure S1. Flow diagram of the literature search and publication selection process for the narrative review
