# Supplementary material for: Cold Atmospheric Plasma and Plasma-Activated Water in Meat Processing: Microbial Safety, Molecular Transformations, Quality Changes, and Analytical Assessment
Source: Molecules. 2026 Oct 15;31(20):3697. doi: 10.3390/molecules31203697 (PMC13707862; doi:10.3390/molecules31203697)
Supplement: Supplementary file 1 [file molecules-31-03697-s001.zip › molecules-4585610-supplementary/Supplementary_Table_S1_Search_Strategies.pdf]

Supplementary Table S1. Database-Specific Search Strategies

Cold plasma and plasma-activated water in meat and meat products

Search period: January 2026 to 20 August 2026 (final search date). **Primary publication period:** 1 January 2016 to 20 August 2026. **Language:** English. **Publication type:** peer-reviewed original research articles. Earlier foundational studies were considered separately for methodological, mechanistic, or historical context.

Database	Field(s)	Limits	Records retrieved
Scopus	TITLE-ABS-KEY	2016–2026; English; Article	n = 133
Web of Science Core Collection	TS (Topic)	2016–2026; English; Article	n = 106
PubMed	Title/Abstract [tiab]	1 Jan 2016–20 Aug 2026; English	n = 108
ScienceDirect	Title, abstract, or author-specified keywords	2016–2026; English; Research articles	n = 1346

S1. Core Conceptual Search Strategy

```
("cold plasma" OR "cold atmospheric plasma" OR "atmospheric cold plasma" OR "non-thermal plasma" OR "nonthermal plasma"
OR "plasma-activated water" OR "plasma activated water" OR "plasma-treated water" OR "plasma treated water" OR PAW)
AND
(meat OR poultry OR chicken OR beef OR pork OR lamb OR mutton OR sausage* OR ham OR "meat product*")
AND
(microbial OR microbiological OR microorganism* OR pathogen* OR decontaminat* OR quality OR physicochemical OR
oxidation OR oxidative OR protein* OR lipid* OR volatile* OR sensory OR color OR colour OR texture OR storage OR "shelf life"
OR shelf-life OR safety OR analytical)
```

S2. Scopus

Search field: TITLE-ABS-KEY (title, abstract, author keywords, and indexed keywords).

```
TITLE-ABS-KEY(
  ("cold plasma" OR "cold atmospheric plasma" OR "atmospheric cold plasma" OR "non-thermal plasma" OR "nonthermal
plasma" OR "plasma-activated water" OR "plasma activated water" OR "plasma-treated water" OR "plasma treated water" OR
PAW)
  AND
  (meat OR poultry OR chicken OR beef OR pork OR lamb OR mutton OR sausage* OR ham OR "meat product*")
  AND
  (microbial OR microbiological OR microorganism* OR pathogen* OR decontaminat* OR quality OR physicochemical OR
oxidation OR oxidative OR protein* OR lipid* OR volatile* OR sensory OR color OR colour OR texture OR storage OR "shelf life"
OR shelf-life OR safety OR analytical)
)
AND PUBYEAR > 2015
AND PUBYEAR < 2027
AND LIMIT-TO(LANGUAGE, "English")
AND LIMIT-TO(DOCTYPE, "ar")
```

Final search date: 20 August 2026. **Records retrieved:** n = [133].

S3. Web of Science Core Collection

Search field: TS (Topic), covering titles, abstracts, author keywords, and Keywords Plus.

```
TS=(
  ("cold plasma" OR "cold atmospheric plasma" OR "atmospheric cold plasma" OR "non-thermal plasma" OR "nonthermal plasma" OR "plasma-activated water" OR "plasma activated water" OR "plasma-treated water" OR "plasma treated water" OR PAW)
  AND
  (meat OR poultry OR chicken OR beef OR pork OR lamb OR mutton OR sausage* OR ham OR "meat product*")
  AND
  (microbial OR microbiological OR microorganism* OR pathogen* OR decontaminat* OR quality OR physicochemical OR oxidation OR oxidative OR protein* OR lipid* OR volatile* OR sensory OR color OR colour OR texture OR storage OR "shelf life" OR shelf-life OR safety OR analytical)
)
```

Refinements: Publication years 2016–2026; language English; document type Article. **Final search date:** 20 August 2026. **Records retrieved:** n = [106].

S4. PubMed

Search field: Title/Abstract [tiab], supplemented with controlled-vocabulary terms where appropriate.

```
(
  ("cold plasma"[tiab] OR "cold atmospheric plasma"[tiab] OR "atmospheric cold plasma"[tiab] OR "non-thermal plasma"[tiab] OR "nonthermal plasma"[tiab] OR "plasma-activated water"[tiab] OR "plasma activated water"[tiab] OR "plasma-treated water"[tiab] OR "plasma treated water"[tiab] OR PAW[tiab])
  AND
  (meat[tiab] OR poultry[tiab] OR chicken[tiab] OR beef[tiab] OR pork[tiab] OR lamb[tiab] OR mutton[tiab] OR sausage*[tiab] OR ham[tiab] OR "meat product"[tiab] OR "meat products"[tiab])
  AND
  (microbial[tiab] OR microbiological[tiab] OR microorganism*[tiab] OR pathogen*[tiab] OR decontaminat*[tiab] OR quality[tiab] OR physicochemical[tiab] OR oxidation[tiab] OR oxidative[tiab] OR protein*[tiab] OR lipid*[tiab] OR volatile*[tiab] OR sensory[tiab] OR color[tiab] OR colour[tiab] OR texture[tiab] OR storage[tiab] OR "shelf life"[tiab] OR shelf-life[tiab] OR safety[tiab] OR analytical[tiab])
)
AND ("2016/01/01"[Date - Publication] : "2026/08/20"[Date - Publication])
AND English[lang]
```

Final search date: 20 August 2026. **Records retrieved:** n = [108].

S5. ScienceDirect

Search field: Title, abstract, or author-specified keywords.

```
("cold plasma" OR "cold atmospheric plasma" OR "atmospheric cold plasma" OR "non-thermal plasma" OR "nonthermal plasma" OR "plasma-activated water" OR "plasma activated water" OR "plasma-treated water" OR "plasma treated water" OR PAW)
AND
(meat OR poultry OR chicken OR beef OR pork OR lamb OR mutton OR sausage OR sausages OR ham OR "meat product" OR "meat products")
AND
(microbial OR microbiological OR microorganism OR microorganisms OR pathogen OR pathogens OR decontamination OR
```

quality OR physicochemical OR oxidation OR oxidative OR protein OR proteins OR lipid OR lipids OR volatile OR volatiles OR sensory OR color OR colour OR texture OR storage OR "shelf life" OR safety OR analytical)

Refinements: Publication years 2016–2026; language English; article type Research article. Truncated forms were replaced by explicit singular and plural variants where required by the interface. **Final search date:** 20 August 2026. **Records retrieved:** n = [1346].

S6. Eligibility Criteria Applied to the Search Results

Inclusion criteria

- Peer-reviewed original research articles published in English.
- Direct cold atmospheric plasma treatment, in-package plasma treatment, or plasma-activated water application.
- Raw, processed, or ready-to-eat meat and meat products, including poultry, beef, pork, lamb, and processed meat matrices.
- For PAW studies, plasma-generated reactive species were transferred to an aqueous medium subsequently applied to meat or meat products.
- At least one microbiological, physicochemical, molecular, oxidative, sensory, or storage-related outcome was reported.
- Sufficient information on the treatment, meat matrix, experimental conditions, or analytical outcomes was available for narrative interpretation.

Exclusion criteria

- Studies focused exclusively on non-meat matrices.
- Medical, biomedical, veterinary therapeutic, or animal-feed applications.
- Packaging-material studies without direct plasma or PAW application to meat.
- Theoretical plasma mechanisms without relevant experimental data for meat products.
- Reviews, conference abstracts, editorials, letters, book chapters, and other non-original or non-peer-reviewed publications as primary evidence.
- Insufficient accessible information to assess treatment conditions or relevant outcomes.
- Duplicate reports of the same experimental dataset; the most complete report was retained.

Contextual sources. Review articles, methodological publications, and earlier foundational studies were used for mechanistic, methodological, or historical context and to identify additional original studies, but were not treated as primary evidence in the narrative synthesis.
